# Enhanced Thermal Stability and Reduced Aggregation in an Antibody Fab Fragment at Elevated Concentrations

**DOI:** 10.1021/acs.molpharmaceut.3c00081

**Published:** 2023-04-11

**Authors:** Cheng Zhang, Jordan W. Bye, Lok H. Lui, Hongyu Zhang, John Hales, Steve Brocchini, Robin A. Curtis, Paul A. Dalby

**Affiliations:** † Department of Biochemical Engineering, University College London, Gower Street, London WC1E 6BT, U.K.; ‡ School of Chemical Engineering and Analytical Science, The University of Manchester, Sackville Street, Manchester M13 9PL, U.K.; § UCL School of Pharmacy, 29-39 Brunswick Square, London WC1N 1AX, U.K.

**Keywords:** fab, aggregation, melting temperature (*T*_m_), entropy change (△*S*_vh_), crowding effect, concentration, dynamic light scattering (DLS), mechanistic model

## Abstract

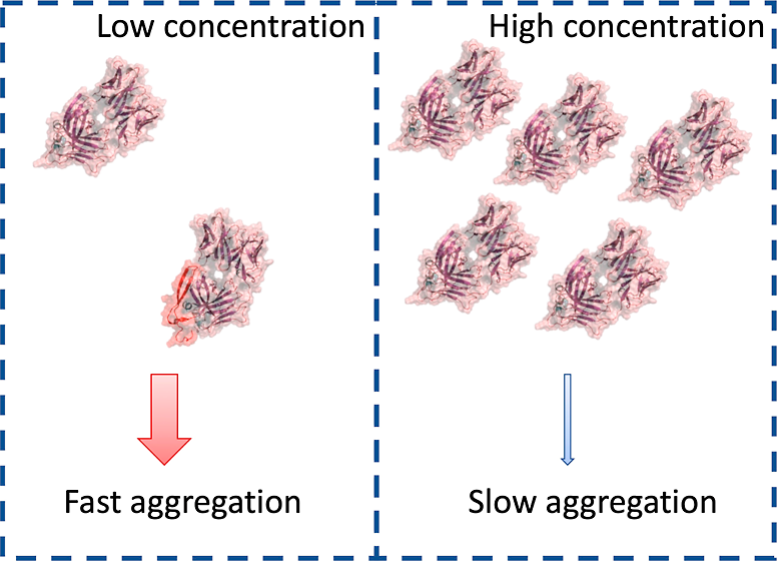

The aggregation of protein therapeutics such as antibodies remains a major challenge in the biopharmaceutical industry. The present study aimed to characterize the impact of the protein concentration on the mechanisms and potential pathways for aggregation, using the antibody Fab fragment A33 as the model protein. Aggregation kinetics were determined for 0.05 to 100 mg/mL Fab A33, at 65 °C. A surprising trend was observed whereby increasing the concentration decreased the relative aggregation rate, ln(v) (% day^–1^), from 8.5 at 0.05 mg/mL to 4.4 at 100 mg/mL. The absolute aggregation rate (mol L^–1^ h^–1^) increased with the concentration following a rate order of approximately 1 up to a concentration of 25 mg/mL. Above this concentration, there was a transition to an apparently negative rate order of −1.1 up to 100 mg/mL. Several potential mechanisms were examined as possible explanations. A greater apparent conformational stability at 100 mg/mL was observed from an increase in the thermal transition midpoint (*T*_m_) by 7–9 °C, relative to those at 1–4 mg/mL. The associated change in unfolding entropy (△*S*_vh_) also increased by 14–18% at 25–100 mg/mL, relative to those at 1–4 mg/mL, indicating reduced conformational flexibility in the native ensemble. Addition of Tween or the crowding agents Ficoll and dextran, showed that neither surface adsorption, diffusion limitations nor simple volume crowding affected the aggregation rate. Fitting of kinetic data to a wide range of mechanistic models implied a reversible two-state conformational switch mechanism from aggregation-prone monomers (N*) into non-aggregating native forms (*N*) at higher concentrations. *k*_D_ measurements from DLS data also suggested a weak self-attraction while remaining colloidally stable, consistent with macromolecular self-crowding within weakly associated reversible oligomers. Such a model is also consistent with compaction of the native ensemble observed through changes in *T*_m_ and △*S*_vh_.

## Introduction

Today, more than 60 antibody-based therapeutics have been licensed to treat various diseases including cancer and infectious and chronic inflammatory diseases.^1,2^ Antibody solutions are often formulated at high concentrations (>50 mg/mL) in their final liquid form for subcutaneous or intramuscular delivery routes, for which aggregation remains as one of the prevailing challenges that affects product efficacy and could also potentially induce serious immunogenicity issues.^3,4^

Potential aggregation mechanisms have been extensively reviewed, with a range of pathways represented by different kinetic models.^5^ Multiple aggregation routes can occur in parallel for a protein under a given set of conditions. Thus, a generic model has not yet been developed to satisfy all protein degradation processes^6^ although generalized models have been developed for some aspects, including the Lumry–Eyring nucleated polymerization model, which assumes equilibrium unfolding.^7^ In this model, aggregation is initialized by reversible unfolding and oligomerization until a nucleation point is reached. Irreversible chain polymerization through the addition of monomers then follows. This model has also been extended to include aggregate–aggregate condensation.^8^ Monomer addition and aggregate coalescence can often propagate simultaneously, so it is important to identify the key influential factors to understand the aggregation growth pattern for a typical protein. Secondary aggregation processes have been identified, for example, in the aggregation of actin, collagen, and sickle hemoglobin, which were characterized as nucleation-controlled, but with specific secondary polymer growth pathways, namely fragmentation, heterogeneous nucleation, and lateral growth.^9^

Recently, a global fitting strategy has been developed to yield compatible kinetic models with defined microscopic processes.^10^ Scaling exponents derived from monomer loss half-time plots were used to indicate the involvement of more complex processes, including 2-step elongation and 1-step or 2-step secondary nucleation and fragmentation.

Protein aggregation stems essentially from either the conformational instability of the native state, or through colloidal instability, or a mix of the two.^11−13^ Conformational instability can result in formation of the globally unfolded state, partially unfolded states, or near-native states that may even be considered simply as part of the dynamic native structure ensemble.^13−17^ Colloidal instability is the result of favorable (net-attractive) self-interaction between native, near-native, partially unfolded, or unfolded states.^18,19^ Both of these mechanisms can be promoted through external stresses in solution and/or at surface interfaces.^20^ To mitigate aggregation, both the intrinsic protein properties (e.g., surface charge, folding energy)^21,22^ and extrinsic solution characteristics (e.g., pH, excipient, concentration, surface chemistry)^23,24^ could be regulated to minimize aggregation. For example, these modifications can aim to fine-tune various controlling factors for aggregation kinetics, including solubility,^25^ diffusion/viscosity,^26^ and protein–protein interactions,^27^ that would enhance the solution characteristics and maintain proteins in their native states.

Previously, the effects of mutations, on the aggregation rate of an A33 antibody fragment (Fab), were studied at 1–8 mg/mL, in which monomer-loss rate orders of around 1 were observed, and the increased concentration resulted in a reduced aggregation proportion for all the variants examined.^28^ In the present study, we explore a far wider range of protein concentrations (0.05 to 100 mg/mL) for the wild-type Fab, to investigate the factors that affect the rate order at a higher concentration (>50 mg/mL), and shed light on the potential aggregation pathways and mechanisms leading to the unexpected protein concentration dependence.

## Materials and Methods

### Fab Production, Thermal Stability Analysis, and Aggregation Kinetics

For the expression from *E. coli* strain W3110, purification using protein G chromatography, gel filtration, and buffer exchange was carried out as previously reported,^16^ except that a Biostat Cplus 30L fermenter (Sartorius Stedim, UK) was used to produce a larger quantity of Fab. Protein concentrations were determined by UV–vis absorbance at 280 nm using an extinction coefficient of 1.4 cm^–1^ mL mg^–1^ (66,329 mM^–1^ cm^–1^) and also confirmed over the concentration range by the linear relationship with SEC peak areas.

The thermal stability analysis and aggregation kinetics were carried out as reported previously,^28^ with 0.05 to 100 mg/mL Fab, at 20 mM sodium citrate, pH = 4, and NaCl added to a total ionic strength of 200 mM. Aggregation kinetics were determined from the rates of monomer loss for up to 480 min, with monomer fraction determined by SEC-HPLC as previously. The SEC-HPLC injection volume was reduced to 1 μL for 25–100 mg/mL samples so as to not exceed the detection limit of the instrument. All monomer loss values were expressed as % monomer retained, by comparison to a standard curve using undegraded Fab. All measurements from degradation kinetics experienced a “dead time” of approximately 2 min between sampling and quenching by cooling prior to SEC analysis.

Monomer loss kinetics were curve-fitted to an exponential function, derived from first-order kinetics
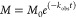
1where *M*_0_ is the initial monomer concentration, *k*_obs_ is the rate constant, *M* is the monomer retention normalized from 0 to 1, and *t* is the incubation time. The first derivative of eq 1
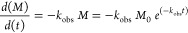
2was used to obtain the initial aggregation rate as *M*_0_*k_obs_ when *t* = 0.

Thermal stability was measured from the change in the barycentric mean (BCM) of intrinsic fluorescence, under thermal scanning at 1 °C/step as previously described,^28^ using the UNit (Unchained Labs, Pleasanton, CA, US). The thermal unfolding profiles were fitted to the van’t Hoff equation^29,30^
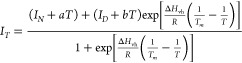
3where
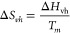
4

to obtain the van’t Hoff thermal parameters Δ*H*_vh_ and Δ*S*_vh_ and the thermal transition midpoint temperature (*T*_m_), where *I*_*T*_ is the BCM at temperature *T*, *I*_*N*_ is the BCM native baseline, *I*_*D*_ is the BCM denatured baseline, *a* is the native baseline slope, *b* is the denatured baseline slope, and *R* is the molar gas constant. The fraction of unfolded protein (*f*_T_) at a certain temperature *T* was calculated from
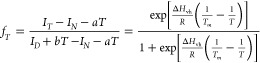
5

### Analysis for the Adsorption Effect

The surfactant Tween 80 (P6474) was purchased from Sigma-Aldrich (Dorset, UK). Aliquots were prepared upon receiving the product and stored at −20 °C. Each aliquot was used to prepare the Tween stock solution, which was then mixed with the stock solutions of Fab and buffer salts to make the final concentration of Tween at 0.01 or 0.1 mg/mL. Afterward, the Fab solution was subjected to thermal incubation as stated previously.

### Viscosity Measurement

The *m*-VROC viscometer (RheoSense, Inc.) was used to measure the dynamic viscosity of protein samples, with a water bath to maintain the flow channel at 65 °C. Stock solutions of Fab, buffer, and excipients were mixed and filtered through 0.22 μm filters prior to the measurement. The filtered sample, contained in a Hamilton 0.5 mL syringe, was loaded onto a syringe jacket. The *m*-VROC measures the pressure drop along an array of sensors when a liquid passes through the cell. The slope (and corresponding *R*^2^ of the fit) is calculated from the pressure drop as a function of distance (essentially shear stress versus shear rate) and is used to calculate the viscosity. During the measurement, shear rates of up to 18,000 s^–1^ were performed with corresponding viscosities recorded. A water sample was always measured as a reference before every protein sample to ensure the cleanness of the flow channel. The reported viscosities were averaged from measurement repeats for which the “Slope Fit *R*^2^” was >0.99. Chemicals were purchased from Sigma-Aldrich (Dorset, UK) with Ficoll 70 (F2878), polyvinylpyrrolidone (PVP40), and dextran 40 (31389) used as crowding agents.

The diffusion coefficient *k*_*d*_ for the bimolecular reaction rate constant was determined based on eq 6,^31^ where *R* is the gas constant and *T* and μ are the absolute temperature and viscosity at 65 °C, respectively.
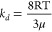
6

### Static and Dynamic Light Scattering (SLS and DLS)

Simultaneous static and dynamic light scattering (SLS and DLS) was measured with a DynaPro NanoStar instrument (Wyatt Technology, UK) for 30 μL sample pipetted into a quartz cuvette (JC-578) and loaded into the pre-heated instrument. DLS acquisitions were time averaged over 5 s intervals and taken at different temperature intervals. DLS readings at a temperature of 25–45 °C were used for determining the protein–protein interaction parameter *k*_D_ based on a cumulant analysis for extracting the diffusion coefficient, while the long-wavelength structure factor *S*0 was extracted from the SLS readings. An isothermal hold for 20–30 min was used at 65 °C in order to follow the time-evolution of aggregate size distributions obtained from a regularization analysis of the intensity auto-correlation function. All analysis was carried out using routines from DYNAMICS software (version 7.8.2.18). Replicates were performed for each concentration from 1 to 100 mg/mL with generally good reproducibility.

## Results and Discussion

### Kinetics of Monomer Loss at a Range of Concentrations

**Figure 1:**
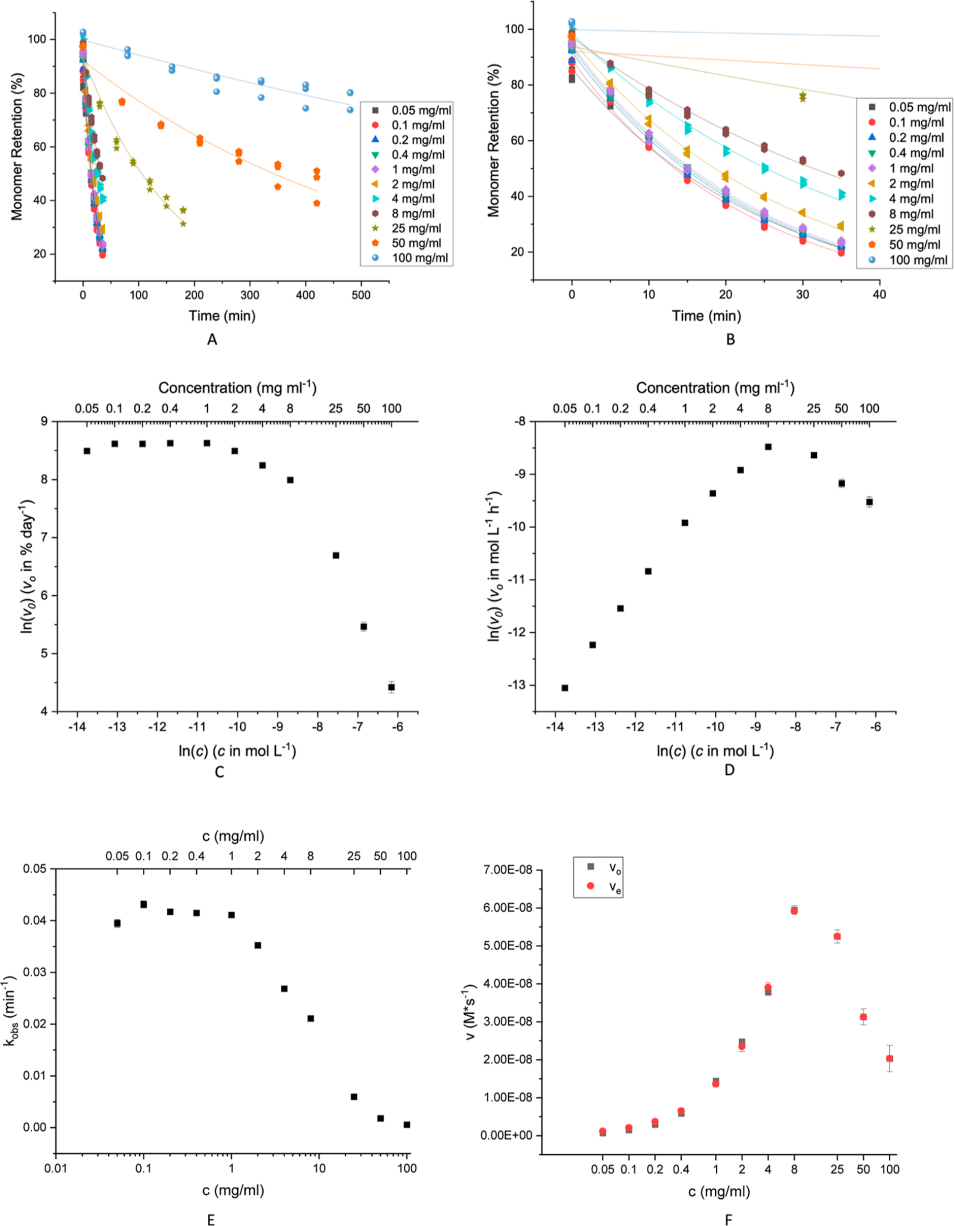
onomer retention and derived aggregation of Fab at concentrations from 0.05 to 100 mg/mL. Samples were incubated in 20 mM citrate, pH = 4, with NaCl to 200 mM ionic strength at 65 °C and analyzed by SEC-HPLC, shown for up to 500 min (A) and with the first 35 min expanded (B). Single exponential curve fitting obtained the relative initial aggregate rate (C), absolute initial aggregation rate (D), and the apparent rate constant *k*_obs_ (E). The rate estimated from the fitted monomer conformation switch model is shown (*v*_e_) along with the observed initial aggregation rates (*v*_o_) (F) (see details in the Supporting Information). Error bars are standard errors (SE) derived from the fitting.

To investigate the influence of the Fab protein concentration on aggregation kinetics, the Fab samples were formulated from 0.05 to 100 mg/mL, then incubated at 65 °C in a thermal cycler, and the retention of the monomer analyzed over time by SEC-HPLC. The monomer retention is shown in Figure 1A,B, and the derived relative and absolute aggregation rate at time 0 is shown in Figure 1C,D, respectively. The data for the apparent rate constant (*k*_obs_) and the observed initial aggregation rates (*v*_obs_) are also shown in Table 1, and Table S1 (Supporting Information).

**Table 1:** Thermal Stability, Aggregation Kinetics, Viscosity, and Diffusion Coefficients at Each Fab Concentration^a^

					monomer loss kinetics					
protein conc. (mg/mL)	*T*_m_ (°C)	*f*_T65_ (%)	Δ*H*_vh_ (kJ mol^–1^)	Δ*S*_vh_ (kJ mol^–1^K^–1^)	*M*_0_	*k*_obs_ (min^–1^)	relative ln(*v*_*0*_) (v_0_ in % day^–1^)	absolute ln(*v*_*0*_) (v_0_ mol L^–1^h^–1^)	viscosity (cP)	diffusion coefficient x10^10^ (L mol^–1^s^–1^)
0.05	70.8 (0.1)	5.5 (0.2)	474 (1)	1.38 (0.01)	0.86 (0.01)	0.039 (0.001)	8.49 (0.03)	–13.05 (0.03)	0.522	1.44
0.1	71 (0.1)	5.0 (0.4)	476 (10)	1.38 (0.03)	0.89 (0.01)	0.043 (0.001)	8.62 (0.02)	–12.23 (0.02)	0.521	1.44
0.2	70.6 (0.1)	5.3 (0.3)	498 (8)	1.45 (0.02)	0.92 (0.01)	0.042 (0.001)	8.62 (0.02)	–11.54 (0.02)	0.521	1.44
0.4	70.8 (0.1)	4.0 (0.25)	530 (20)	1.55 (0.06)	0.94 (0.01)	0.041 (0.001)	8.63 (0.01)	–10.84 (0.01)	0.518	1.45
1	72.6 (0.3)	3.1 (0.5)	442 (2)	1.28 (0.01)	0.95 (0.01)	0.041 (0.001)	8.63 (0.02)	–9.92 (0.02)	0.524	1.43
2	71.6 (0.1)	4.9 (0.2)	435 (1)	1.26 (0.01)	0.96 (0.01)	0.035 (0.001)	8.50 (0.01)	–9.36 (0.01)	0.524	1.43
4	70.1 (0.3)	9.0 (1.1)	438 (1)	1.28 (0.01)	0.99 (0.01)	0.027 (0.001)	8.25 (0.02)	–8.92 (0.02)	0.524	1.43
8	73.6 (0.1)	1.9 (0.1)	447 (1)	1.29 (0.01)	0.97 (0.01)	0.021 (0.001)	7.99 (0.02)	–8.48 (0.02)	0.530	1.41
25	77.2 (0.1)	0.18 (0.01)	511 (1)	1.46 (0.01)	0.94 (0.02)	0.0060 (0.0005)	6.69 (0.05)	–8.64 (0.05)	0.570	1.32
50	78.4 (0.05)	0.099 (0.002)	511 (1)	1.45 (0.003)	0.92 (0.02)	0.0018 (0.0001)	5.47 (0.08)	–9.17 (0.08)	0.629	1.19
100	79.9 (0.3)	0.036 (0.002)	530 (14)	1.50 (0.04)	1.00 (0.01)	0.0006 (0.0001)	4.4 (0.1)	–9.5 (0.1)	0.800	0.937

a Initial velocities (in % day^–1^) are calculated as *v*_0_ = 100 M_0_*k*_obs._ Standard errors of the mean (SEM) are shown in parentheses.

As shown in Figure 1A and B, increasing the concentration of Fab greatly mitigated the rates of monomer loss. Between 50 and 70% of the monomers aggregated irreversibly at concentrations between 0.05 and 8 mg/mL in the first 30 min, whereas samples at 25 mg/mL lost less than 30%, and those at 100 mg/mL lost less than 5% of monomers.

The concentration effect on aggregation could be captured more clearly using the relative initial rates of monomer loss (% day^–1^) in Figure 1C. This relative “aggregation” rate increased slightly from 0.05 to 1 mg/mL, then declined slowly from 2 to 8 mg/mL, and finally saw nearly 1 order of magnitude reduction as the Fab concentration doubled from 25 to 50 mg/mL and then again from 50 to 100 mg/mL. At 100 mg/mL, the Fab gave a relative aggregation rate, *v*_0_ of 83% day^–1^, which was 67-fold slower than that at 1 mg/mL.

It was also useful to compare the absolute initial rate of monomer loss in mol L^1^ h^1^. As shown in Figure 1D, this increased by almost 1 order of magnitude as the concentration doubled from 0.05 to 8 mg/mL. The increase plateaued at 8 mg/mL, and then, the rate of monomer exhibited a maximum at 25 mg/mL, and further dropped at 100 mg/mL to a similar level (ln(*v*_0_) = −9.52) to that at 2 mg/mL (ln(*v*_0_) = −9.36). The rate order at any concentration was obtained from the slope of ln(*v*_0_) versus ln(*c*) as shown in Figure 1D. It changed from approximately 1 (0.05–1 mg/mL) to −1.1 (100 mg/mL) (Table S2, Supporting Information). However, a negative rate order is not possible and so must be attributable to a change in the mechanism, or presence of multiple pathways, that affected the apparent kinetic constant at increased concentration.

The next aim was to investigate the molecular mechanism of aggregation further to better inform the choice of mechanistic kinetic models that can fit and potentially explain the data. Therefore, the thermal stability, surface adsorption, viscosity, and average radius of the Fab in formulations were characterized.

### Thermal Stability Analysis

**Figure 2:**
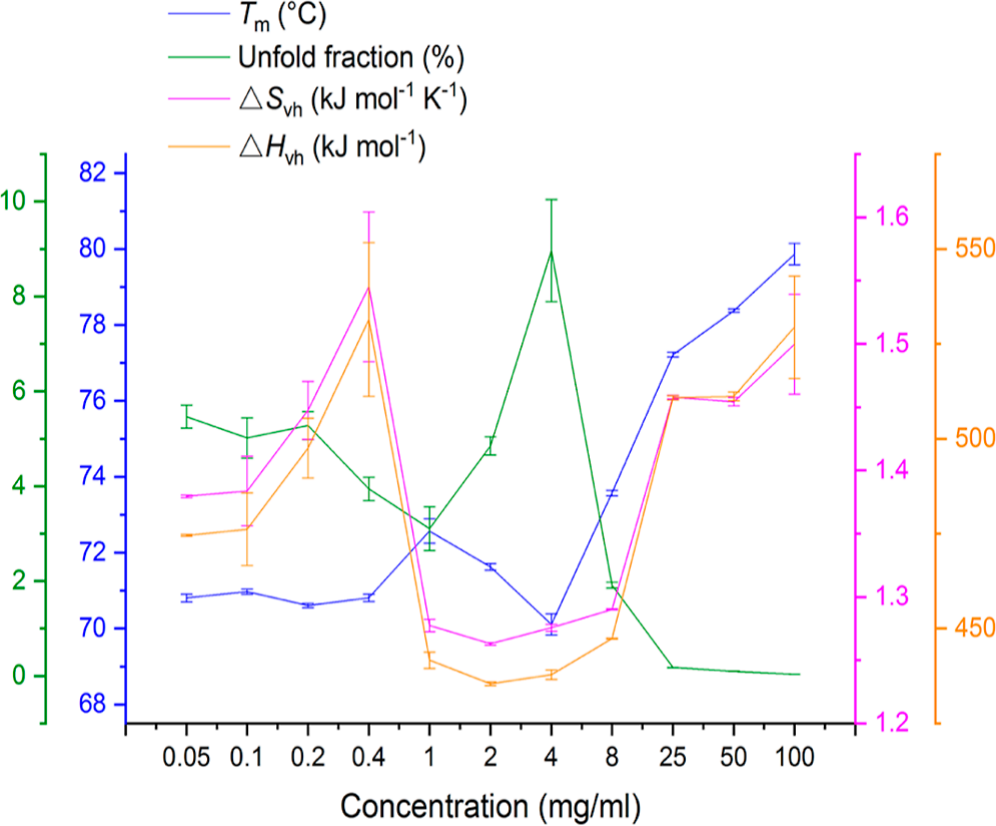
Thermal denaturation midpoint temperature (*T*_m_), unfolded fraction at 65 °C, and enthalpy and entropy changes at the midpoint of transition (Δ*H*_vh_ and Δ*S*_vh_) were plotted against Fab concentrations from 0.05 to 100 mg/mL in 20 mM sodium citrate, pH = 4, and NaCl to 200 mM ionic strength. All error bars were standard error of the mean (SEM).

The Fab was formulated at 0.05 to 100 mg/mL and subjected to thermal ramping from 20 to 90 °C to characterize its thermal stability. The Fab unfolding behavior was captured by the shift of the BCM of intrinsic protein fluorescence (Figure S1, Supporting Information). The van’t Hoff thermal parameters Δ*H*_vh_ and Δ*S*_vh_ and thermal denaturation midpoint temperature (*T*_m_) were obtained by fitting the BCM to the van’t Hoff equation, and these are summarized in Table 1 and also plotted together in Figure 2 with the fraction of unfolded protein (*f*_65_) calculated at a temperature of 65 °C used for aggregation kinetics.^28^

The apparent *T*_m_ values remained within a narrow range (70.6 to 71 °C) from 0.05 to 0.4 mg/mL, while the Δ*S*_vh_ (and Δ*H*_vh_) increased slightly, and the fraction unfolded, *f*_65_ decreased slightly. The *T*_m_ values for Fab are known to have been derived from a convolution of thermal unfolding and the rapid irreversible aggregation of thermally denatured protein in these experiments^16^ and so the increase in Fab concentration in this range, presumably did not have a significant impact on the kinetics of heat-induced aggregation. Higher values of Δ*S*_vh_ relate to a more cooperative transition, which can result from a more compact native ensemble and/or fewer intermediate states populated during denaturation and subsequent aggregation. Thus, the thermal stability of Fab was slightly improved in going from 0.05 to 0.4 mg/mL, in terms of a more compact native state, but with no impact on the *T*_m_ values, suggesting a small (if any) increase in either solvent surface tension, molecular crowding, or a protective (non-aggregation-prone) self-interaction, under these dilute conditions.

The *T*_m_ between 1 and 8 mg/mL deviated sharply away from the general trend. It increased slightly to 72.6 °C at 1 mg/mL, from 71 °C at 0.4 mg/mL, on an upward trend that is seen to continue at 25–100 mg/mL. However, it began to decrease at 2 mg/mL, reaching a minimum of 70.1 °C at 4 mg/mL, before increasing again sharply at 4 to 25 mg/mL, by approximately 7 °C. These changes were accompanied by a significant decrease in Δ*S*_vh_ at 1–8 mg/mL to around 1.27 kJ mol^–1^ K^–1^, before it again increased back to previous levels with 1.50 kJ mol^1^ K^–1^ at 100 mg/mL. This trend was also reflected in the increased *f*_65_, particularly at 2 mg/mL and 4 mg/mL.

The non-monotonic behavior implied the coexistence of at least two opposing mechanisms at different concentration ranges. As the concentration increased, the general trend was an increase in *T*_m_, an increase in Δ*S*_vh_, and a decrease in *f*_65_. This is consistent with a gradual increase in either solvent surface tension, molecular crowding, or a protective self-interaction, leading to a more compact native ensemble and/or fewer intermediate states during denaturation, which is then less prone to aggregation from partially unfolded states. The second effect led to the observed spike in *f*_65_ at 2–8 mg/mL and the associated dip in *T*_m_ and Δ*S*_vh_. At this intermediate concentration range, the absolute aggregation kinetics were still increasing (Figure 1), as would be expected from the increased collisional frequency between molecules. However, it was the same concentration range over which the rate constant *k*_obs_ and the relative initial rate began to decrease. In particular, *k*_obs_ decreased by the greatest amount over 2–8 mg/mL, before decreasing more modestly at >8 mg/mL (Figure 1E). The loss of apparent unfolding cooperativity (lower Δ*S*_vh_) could reflect a less compact native state due to partial unfolding. This was calculated as the increased fraction unfolded when assuming only a simple two-state transition. However, these observations are also consistent with the population of additional folded states such as alternative native-like conformations, dimer or other soluble oligomer, in equilibrium with the native monomer. Both possibilities broaden the range (and associated stabilities) of species from which thermal unfolding/aggregation can occur.

Further increase in the protein concentration at above 8 mg/mL, stabilized the native ensemble into a more compact form and also suppressed the formation of aggregates to give a higher denaturation temperature, despite the increased collisional frequency. Thus, the *T*_m_ increase at 8 mg/mL is associated with the point at which the relative aggregation rate began to decrease, and the absolute aggregation rate peaked (Figure 1D).

Samples at 8 mg/mL and above had distinctly elevated *T*_m_ values, high Δ*S*_vh_, reduced two-state fraction unfolding (<0.2% above 25 mg/mL), and also significantly decreased aggregation kinetics at 65 °C. The observations of increased *T*_m_ and decreased fraction unfolding indicate the stabilization of the native state relative to the unfolded state at higher protein concentrations. However, one additional feature that would lead to the high Δ*S*_vh_ would be the suppression of partial unfolding at 65 °C, i.e., compaction of the average native state through fewer conformations within the ensemble, resulting in inhibition of aggregation. This could arise for example through a more crowded environment at higher protein concentrations. Another possibility is that the increased *T*_m_, and gradually increasing Δ*S*_vh_ at 8–100 mg/mL was due to a greater population of a protective dimer/oligomer, such that the association into a dimer would increase the stability against unfolding of the monomers. However, Δ*S*_vh_ in this case would only increase in the particular scenario whereby dissociation back to monomers and their unfolding occurred cooperatively, which seems unlikely. Such oligomeric species were also not observed by SEC and so if present, they would either have to be at levels below the limit of detection or otherwise rapidly dissociated upon dilution onto the column.

As molecular crowding at higher protein concentrations could also potentially play an important role in minimizing the native Fab flexibility and the apparent inhibition of aggregation, we investigated this assumption using crowding agents. In addition, the viscosity of the solution at elevated protein concentrations may potentially have suppressed aggregation due to a decreased diffusion rate; and therefore, this was also investigated.

### Does Diffusion Rate Affect the Kinetics of Monomer Loss?

**Figure 3:**
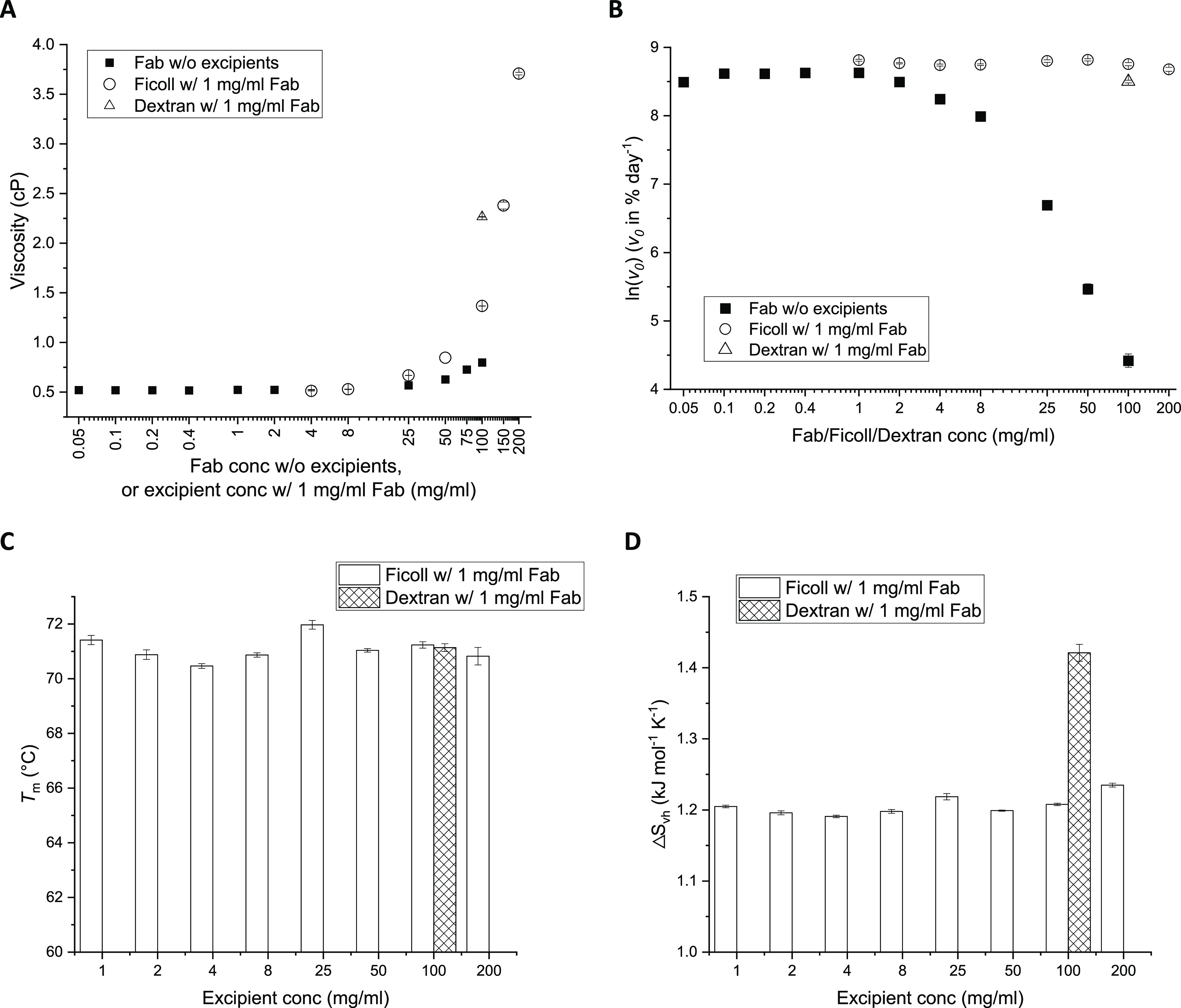
iscosity (A), aggregation rate (B), *T*_m_ (C), and △*S*_vh_ (D) of Fab with and without excipients. Samples were formulated at the same pH and buffer conditions as they were in the thermal stability analysis and aggregation kinetics for Fab without any excipients (black filled square) and Ficoll (hollow circle) and dextran (hollow triangle) with 1 mg/mL Fab. Fab and Ficoll samples were formulated at various concentrations, while dextran samples only at 100 mg/mL. Error bars are standard error of the mean (SEM) for Figure (A,C,D), and are standard errors (SEs) derived from curve fitting for Figure (B).

Equations 1 and 2 assume that the rate of monomer loss follows first-order decay kinetics. Our previous study of Fab A33^28^ has found that the kinetics fitted well with monomolecular reactions from native-like states, but with possible contributions from bimolecular diffusion-limited reactions. Therefore, if bimolecular diffusion-limited reactions were important, then it would be reasonable to expect a role for increased viscosity and decreased diffusion rate, in lowering the aggregation rates at elevated Fab concentrations (Figure 1). The viscosity of the Fab formulations was measured (Figure 3A) and found to remain at around 0.52 cP from 0.05 to 8 mg/mL, before a small increase to 0.80 cP at 100 mg/mL (Table 1).

To test the impact of these higher viscosities but generated independently of protein concentration, the crowding agents Ficoll and dextran were used to create viscous solutions of 1 mg/mL Fab. While the crowding agents led to much higher viscosities than the Fab alone at 1 mg/mL (Figure 3A), this did not affect their rates of monomer loss compared to the 1–2 mg/mL Fab without any excipients (Figures 3B and S2 in Supporting Information). Therefore, the addition of viscosity-modifying crowding agents had no evident effect on the aggregation rate, even with a 7-fold increase in solution viscosity. This indicated that the aggregation at 65 °C at the lower Fab concentration range was not diffusion-limited by a second-order reaction but was indeed rate limited by a unimolecular reaction, consistent with the rate order of 0.91 ± 0.04 derived from the initial slope (0.05–8 mg/mL) of Figure 1D. The increase in viscosity observed at above 8 mg/mL is potentially, though not necessarily due to self-association behavior such as transient interactions or the formation of small oligomers in solution at these concentrations.

The decrease in the rate of monomer loss at higher concentrations, showing an apparently negative rate order, was not simply due to an increase in viscosity. Furthermore, as Ficoll and dextran are also crowding agents, their addition did not decrease the rate of monomer loss through any impact on the conformational flexibility of the native protein. Thus, molecular crowding by “hard spheres”^32^ i.e., agents that occupy volume without interacting with the protein, was ruled out. This was further confirmed by showing that the addition of crowding agents also did not alter the thermal stability of 1 mg/mL Fab. The *T*_m_ remained between 70 and 72 °C with Ficoll concentrations ranging from 1 to 200 mg/mL and also with Dextran at 100 mg/mL (Figure 3C). Their Δ*S*_vh_ values also remained constant at all Ficoll concentrations (Figure 3D), although 100 mg/mL dextran increased the Δ*S*_vh_ to 1.42 kJ mol^–1^ K^–1^. Thus, the earlier increases in *T*_m_ and Δ*S*_vh_, which lowered the aggregation rate at higher protein concentrations (Figures 1 and 2), were more likely the result of molecular crowding induced by self-interaction of the protein, at least transiently, to either favor the formation of more compact monomers or otherwise weakly associated dimers or oligomers.

### Does Surface Adsorption Affect the Monomer Loss?

**Figure 4:**
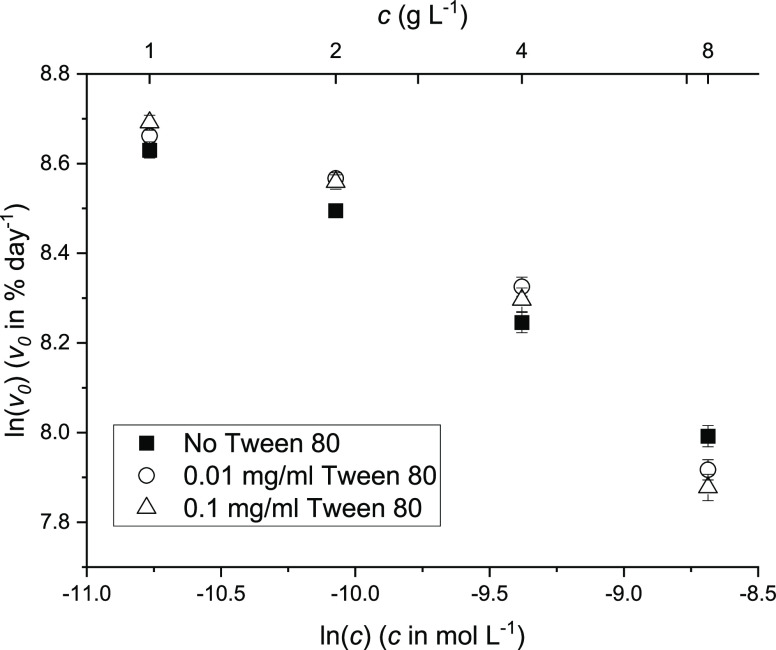
elative aggregation rate of Fab at concentrations from 1 to 8 mg/mL, with or without the presence of Tween 80. Samples were incubated in 20 mM citrate, pH = 4, with NaCl to 200 mM ionic strength at 65 °C and analyzed by SEC-HPLC (see Figure S3, Supporting Information).

We investigated whether the saturation of a finite number of container surface or air-bubble binding sites through non-specific adsorption could explain the overall slowing of the absolute rate of monomer loss at above 8 mg/mL (Figure 1C). This assumed a finite surface area for adsorption of proteins onto the container surface (i.e., liquid–solid interface)^33^ or liquid surface (i.e., liquid–air interface)^34^ that accumulated Fab molecules and promoted their aggregation. At low concentrations, the adsorption sites would not yet be saturated, so aggregation rates could increase linearly with the concentration. At higher concentrations, the effective adsorption sites would become occupied, leaving the majority of the monomers to remain non-aggregating in the bulk aqueous phase. To test this hypothesis, we used the surfactant^35^ Tween 80 on the aggregation of 1–8 mg/mL Fab. Tween concentrations were deliberately selected to be both above and below its critical micelle concentration which is 0.0161 mM (i.e 0.0211 mg/mL) at 65 °C^36^ so that the Tween would primarily adsorb to the liquid–solid and liquid–air interfaces without creating micelles that interfere with protein–protein interactions. We found that the addition of Tween 80 at 0.01 or 0.1 mg/mL did not significantly alter the aggregation rates at any Fab concentration (Figure 4). Therefore, the adsorption of protein to surfaces was unlikely to have been a major factor in the observed rates of monomer loss for Fab.

This result was in line with a previous study,^37^ in which no correlation was observed between the protective effects and the critical micelle concentration of Tween 80. As there was no agitation during the thermal incubation of Fab solutions, the presence of air–liquid interfaces was minimized and so was already unlikely to greatly influence Fab aggregation kinetics through this mechanism.^34^ To conclude, the comparable aggregation rates observed with or without the presence of Tween indicated that the concentration-dependent Fab aggregation kinetics were not due to surface adsorption effects.

### DLS to Measure the Average Radius, Particle Size Distribution, and *k*_D_

Finally, we investigated the role of higher-order aggregate species in the kinetics of monomer loss, as these might be expected to have a concentration-dependent behavior. For example, a specific self-interaction, e.g., dimerization, that is increasingly present at higher Fab concentrations, could potentially be protective against further aggregation. Alternatively, coalescence of oligomers into larger particles at higher protein concentrations might remove the total concentration of nucleation sites from solution and create a slowing of the aggregation rate.

The aggregation pathway kinetics at different Fab concentrations were probed by DLS during the incubations at 65 °C. It should be noted that DLS signal intensity is disproportionately sensitive to larger diameter (*d*) particles as a function of *d*^6^. As a result, the average radii and their kinetics will be skewed toward even small populations of large particles as they form. Nevertheless, it is useful to track whether the protein concentration impacts the average size of particles formed.

**Figure 5:**
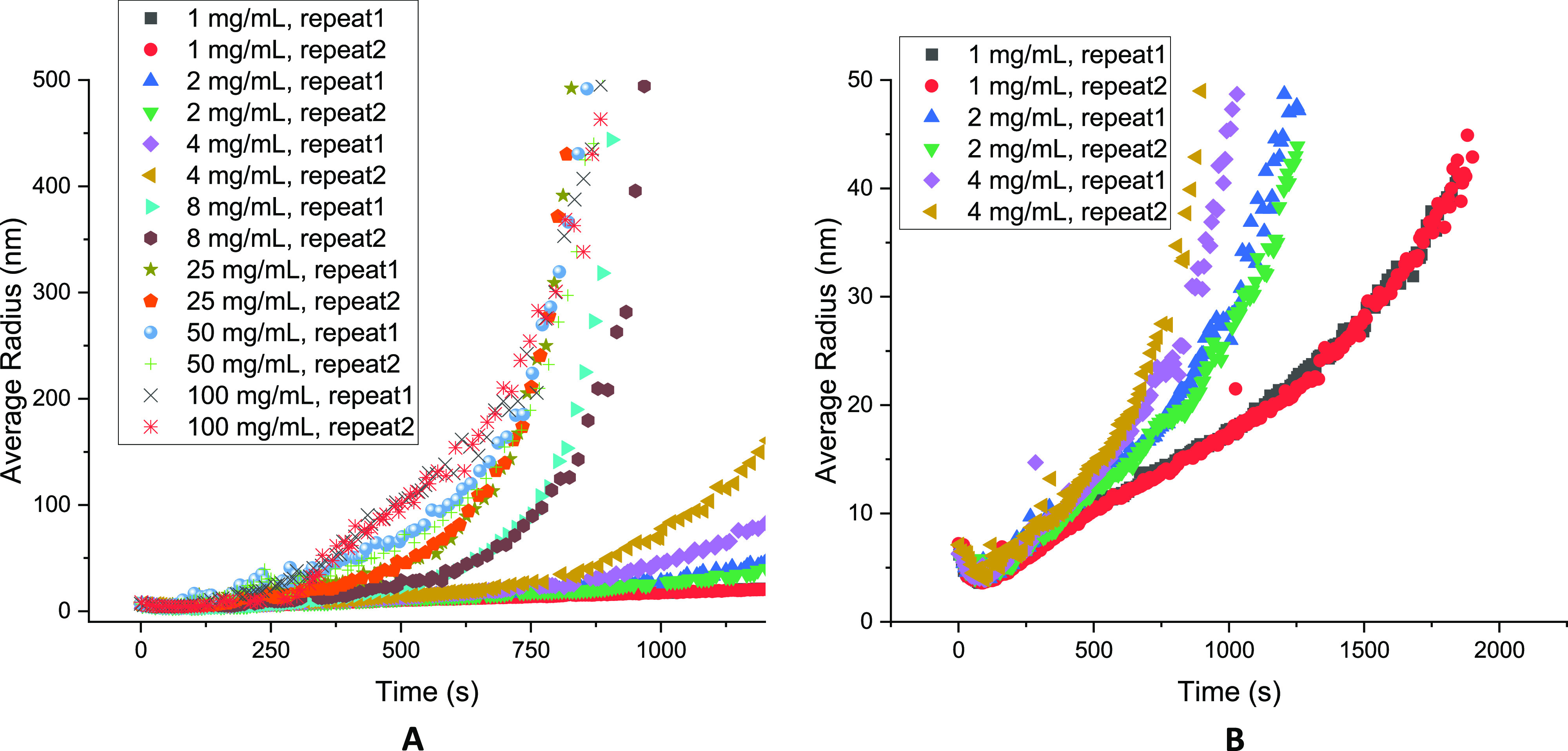
Evolution of the average radius of Fab during the thermal incubation. The average radius was measured by DLS at 65 °C, pH = 4, and 200 mM ionic strength. Duplicates were performed for each concentration. (A) Average radius of all the concentration during the thermal incubation, with samples of 1–4 mg/mL shown in (B).

The change in the average radius over time (Figure 5A) showed two distinct groupings by the Fab concentration. At the lower concentrations of 1 to 4 mg/mL, their radii remained at less than 50 nm for the first 1000 s (Figure 5B). This was not surprising as the protein monomers would take longer to form larger aggregates at diluted conditions. Samples at higher Fab concentrations of 8 to 100 mg/mL, witnessed a sharp increase in radii to more than 500 nm in the first 1000 s. The rank order of the radius in the initial 700 s, corresponded to protein concentrations (i.e., 25 mg/mL < 50 mg/mL < 100 mg/mL). These then diverged into a different rank order at above 700 s, where the radius of the 25 mg/mL samples became the greatest (Figure 5A). This coincided with the maximum absolute aggregation rate at 8–25 mg/mL (Figure 1D).

The average radii increased with an apparent single exponential growth (individual curves are also shown for clarity in Figure S4, Supporting Information), except at 100 mg/mL where an additional intermediate growth phase from 20 to 130 nm, was evident between 250 and 600 s, making the average radius clearly greater than for any other concentration during that period. However, the unexpectedly lower rate of monomer loss under the same conditions (Figure 1) and also the lower rate of radius growth after 600 s, compared to 8–50 mg/mL formulations, suggested that the initial rapid formation of large particles became muted by a decrease in the available nuclei for irreversible aggregation. Notably, this switch in order at 600 s corresponded to a point at which only <5% of monomer loss had yet occurred at 50–100 mg/mL (Figure 1A). Thus, it appeared that the available nuclei in the starting material were disproportionately lower at the higher protein concentrations and that those available were already consumed in a rapid “burst” phase.

**Figure 6:**
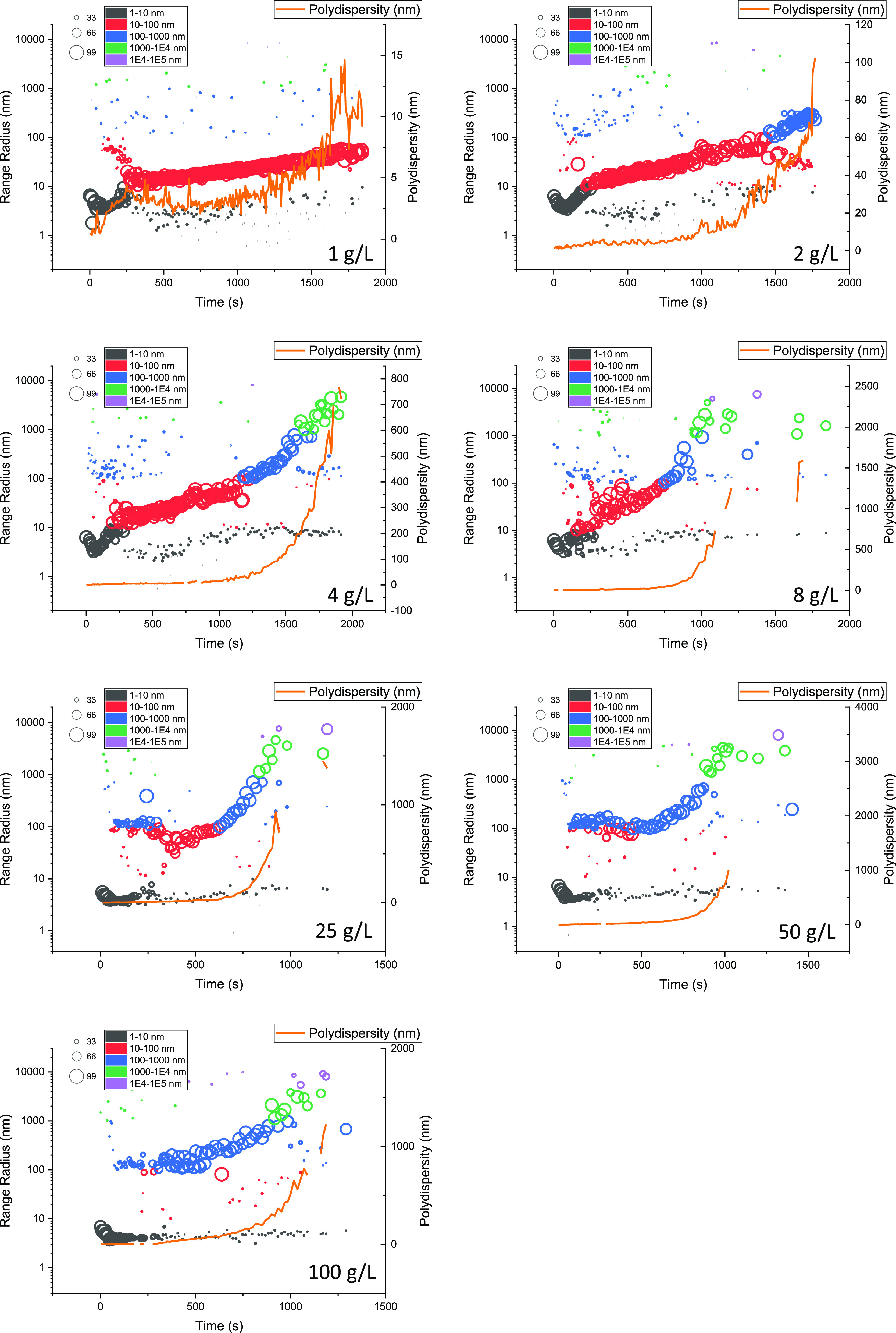
Evolution of the particle size distributions during the incubations at 65 °C, for each Fab A33 concentration. Left *Y* axis: the aggregate radii were color-labeled for different sizes, and population size mapped with regard to their percent intensity. Right *Y* axis: the polydispersity represents the spread of the particle size. The missing points are when the instrument could not capture the polydispersity due to the sampling noise.

To further investigate the aggregation pathway at different concentrations, the particle size distributions were also plotted as a function of time as shown in Figure 6. Aggregate radii were mapped according to their percent intensities, which are proportional to the sixth-power of their diameters (*d*^6^). Furthermore, it should be noted that DLS does not easily resolve particles that differ by less than 10-fold in radius. In general, the monomer population initially formed aggregates that gave average radii of 10–20 nm, which then gradually increased in size through a continuous growth model. These reached larger aggregate sizes for Fab solutions at higher concentrations. The samples at 25–100 mg/mL formed much larger oligomers of 100–1000 nm within the initial 250 s, such that they could be detected as a population separate from the monomers. The initial population at around 4–5 nm was also visible for longer by DLS at the higher concentrations, indicating that the main aggregate forms remained mostly much larger and therefore easily resolved from the monomer by DLS. Overall, the faster production of large aggregates and larger particle size reached at higher protein concentrations was not obviously linked to the slower rates of monomer loss. However, this difference was most likely due to the greater sensitivity of DLS toward larger particles which does not reflect the molar or mass changes in the monomer.

DLS was also used to characterize the protein–protein interaction parameter *k*_D_ under the same conditions used at 200 mM ionic strength (Figure S5, Supporting Information). A linear relationship between the hydrodynamic radius and concentration was obtained only at temperatures below 45 °C and extrapolated to derive the hydrodynamic radius, *R*_h0_, at infinite dilution. Data could not be used at higher temperatures due to rapid aggregation. The *R*_h0_ increased from 3.16 nm at 25 °C to around 3.33 nm at 45 °C, suggesting that the Fab was marginally expanded with a small transition at approximately 29 °C, a possible indication of N populating alternative conformations at the higher temperature. The *k*_D_ remained at around −2 mL/g, indicating a weak attractive self-interaction, but where the Fab remained colloidally stable throughout the temperature range of 25–45 °C. SLS measurements of the long wavelength structure factor *S*0 at 25 °C over the full protein concentration range were well-captured using an adhesive hard-sphere model (Figure S6, Supporting Information) confirming the presence of weak multi-body attractive interactions at high protein concentrations, but no reversible self-association was detectable. Measurements could not be accurately obtained at 65 °C, but the trend was likely to remain the same or become more strongly self-interacting.

### Kinetic Modeling to Elucidate the Aggregation Mechanism

The kinetics clearly showed that an increased concentration (more than 25 mg/mL) of Fab A33 could greatly suppress the rate of Fab A33 aggregation. Several kinetic models were evaluated for their ability to fit to the observed concentration dependence, as detailed in the Supporting Information. It was found that the kinetics could be fitted well using the Finke–Watzky model (Figure S7, Supporting Information), but the rate constants obtained remained concentration-dependent, whereby changes in the relative rate constants for the nucleation and elongation phases enabled a good fit to the data. However, such a model does not explain the concentration-dependent behavior.

We next examined a broad range of alternative kinetic models (Figures S8–S16, Supporting Information) that would better account for the observed concentration dependence. It was hypothesized that a concentration-dependent reversible formation of higher-order oligomers of size n could decrease the availability of monomers or nuclei from the pool that would otherwise lead to further nucleation or aggregate elongation. As discussed below, this hypothesis was insufficient on its own to explain the kinetics observed, and additional inhibitory mechanisms were also required. The best fits to the data, as determined from linear correlations between observed and predicted absolute rates for the monomer loss kinetics, were obtained with (i) a “variable reaction order″ (model 2); (ii) a “two-state monomer conformation switch″ (model 3); (iii) an “off-pathway dimer D and oligomers″ (model 7); and (iv) an “off-pathway dimer D as an inhibitor” (model 8). These are tabulated in Table S3 (Supporting Information).

The “variable reaction order″ (model 2) aimed to adjust the rate order as the concentration increases by introducing a scaling function. This simulated the overall profile (*R*^2^ = 0.94), but it was also clear that it failed to account for the leveling off of the rate at higher concentrations, where the model instead descended asymptotically toward zero.

The “two-state monomer conformation switch″ (model 3) aimed to introduce a conformational transition from aggregation-prone at low concentrations to non-aggregation-prone at high concentrations, by analogy with two-state reversible chemical or thermal denaturation transitions. The fitting converged well with *R*^2^ = 0.997, implying that a two-state conformational equilibrium was a good proxy for macromolecular self-crowding effects at higher concentrations.

A range of off-pathway oligomer models were also examined using dimers to simplify the oligomer formation due to its mathematical closed-form solution. Assuming aggregation rate orders of 1 (model 4), 2 (model 5), and variable m (model 6) for the remaining free monomers could not recreate the observed kinetics. The off-pathway dimer D and oligomers model 7 (*R*^2^ = 0.92) was plausible, though with five critical parameters, and not backed up by any evidence of dimer or soluble oligomers in the SEC data. The dimer inhibitory model 8 (*R*^2^ = 0.99) only had three parameters and is plausible. It also was not supported by any evidence of a dimer in the SEC data, although the parameters obtained estimated the dimer concentration to reach 0.1% of the total protein.

In model 3, a two-state monomer conformation switch was assumed, for example, due to macromolecular self-crowding, that shifts the equilibrium from a low-concentration monomer population *c*’ that is essentially an aggregation-prone native-like state (N*) to a higher concentration monomer population *c*’’ that is a non-aggregation-prone closed form (*N*). This model used six parameters overall, but only two define the sigmoidal transition. The fit gave estimates for the transition midpoint concentration *c*_50__=_ 2 × 10^–4^ M ± 50% (i.e., 9.4 mg/mL), and the reaction rate order *m*’ = 0.82 ± 0.07. Thus, the fraction of monomer species in solution, *c*’ or N*, that were aggregation-prone, ranged from 1 in every 1.2 million molecules at 0.05 mg/mL to 1 in 1.37 million at 4 mg/mL and then dropped to 1 in 76 million at 100 mg/mL. This can be compared to the average concentration of one in 22,000 molecules, i.e., 0.005%, for the aggregation-prone near-native state estimated in our previous models fitted to the observed aggregation kinetics of Fab variants at 1–8 mg/mL.^28^ The aggregation kinetics of the variants ranged up to 10x faster than for WT Fab, which thus led to the higher predicted population of N*.

Lastly, we attempted to rationalize the relative rates of aggregate growth to aggregate formation using the Lumry–Eyring nucleated polymerization model in which it is assumed that the native monomer folding/unfolding equilibrium is much faster than aggregation rates.^7,8^ Within the model, we assumed that the fraction of monomer as N* is proportional to the fraction of unfolded protein as estimated from the unfolding curves (see Figure 2). Measurements of aggregate size as a function of monomer loss (see Figures S17 and S18) indicated that there is a minimum in the relative rates of aggregate growth to formation at 8 g/L. Within the Lumry–Eyring nucleated polymerization model, this minimum is predicted to occur at 4 g/L when only considering aggregate growth by chain polymerization (monomer addition). Growth by chain polymerization occurs at short times, but the results indicated a crossover at longer times to growth by aggregate–aggregate coalescence for the runs at all protein concentrations. Including the effects of aggregate coalescence might explain the discrepancy between the predicted and measured relative rates of aggregate growth but requires more quantitative measurements of aggregate molecular weight over a larger range of monomer loss fraction, which is beyond the scope of this work.

## Conclusions

The present study showed greatly improved stability of a therapeutic A33 antibody fragment through increasing the protein concentration. A more than 7 °C increase in *T*_m_ and 17% increase in Δ*S*_vh_ was obtained at 100 mg/mL compared to 1 mg/mL, demonstrating a huge improvement in the thermal stability and reduced flexibility. As a result, the aggregation rate at 100 mg/mL was 67 times lower than that at 1 mg/mL, which was a considerable improvement.

To elucidate the stabilizing effect at increased concentrations, surface-mediated and bulk-mediated^20^ aggregation mechanisms were examined. Any adsorption effect was found to be insignificantly modified by the addition of Tween, which excluded the surface-mediated hypothesis. For the bulk-mediated mechanisms, several aggregation processes could interplay in the overall monomer depletion and aggregate growth, including conformational instability, diffusion limitations, macromolecular crowding, and changes in aggregate growth versus fragmentation behaviors. The addition of crowding agents demonstrated little impact on Fab monomer loss kinetics or conformational stability, even where the viscosity was increased up to 7 fold. Thus, while the added crowding agents had comparable molecular weights to the Fab, they did not have the same stabilizing effect as simply increasing the concentration of Fab. Meanwhile, the kinetics indicated that the Fab aggregation at 65 °C was not a second-order reaction, but began with a rate order of 1. Therefore, diffusion/viscosity and protection by volume crowding were not driving the slowing of monomer loss.

Overall, while it appeared that high Fab concentrations led to the fastest initial growth in aggregate particle size as seen by DLS, the more quantitative analysis by SEC showed that the same conditions actually led to a slower rate of monomer loss. While the aggregates grew in size gradually over time, their fraction of the total protein by mass was likely still small due to the biased sensitivity of DLS toward larger particles. Instead, the rate order of 1 for monomer loss kinetics at lower protein concentrations indicated an aggregation rate limited by partial unfolding of remaining native monomers (N) into an aggregation competent form (N*). The existence of such an N* form is already well-documented for Fab A33 using molecular dynamics simulations, small-angle X-ray scattering, and single-molecule FRET measurements and is known to result from destabilization of the *C*_L_ domain under the same conditions that promote aggregation.^17^

Several possible mechanisms can be hypothesized to explain the slowing of monomer loss at higher protein concentrations. A crowding effect was the most likely explanation but not one that could be replicated by adding crowding agents such as Ficoll. Thus, it was more likely to involve a self-interaction between proteins at the higher concentrations, consistent with the negative *k*_D_ values measured by protein-concentration-dependent DLS at lower temperatures and the SLS measurements of *S*0 over the full protein concentration range. Indeed, the best fit of the kinetic data was to a model in which a two-state reversible transition was induced at a particular protein concentration and shifted the monomer from an aggregation-prone form (N*) to a more compact non-aggregation-prone native form *N*. This slowed the aggregation kinetics at higher protein concentrations.

The specific nature of this self-interaction is not determined here and could be consistent with weak or transient interactions between the *N* form that increase the persistence of this form over the N* form. The weak self-association into particles is consistent with the lack of observable soluble oligomers by SEC which would continue to report them as remaining monomers. Simple concentration-dependent formation of a dimer through a specific, reversible, and well-defined single interaction did not model the kinetics so well. It remains possible that the self-interaction of proteins to form the larger particles observed by DLS was involved in driving the transition from aggregation-prone monomers N* to non-aggregation-prone monomers *N*, but this cannot be deduced based on DLS data due to its inability to resolve species well or to make fully quantitative measurements.

This work has significant implications for the development of high-concentration protein formulations. However, it is not yet clear if this stabilizing effect is unique for this Fab species. Investigating this further could therefore bring a potential route for the rational stabilization of other high-concentration proteins into reversible and non-aggregation-prone oligomers.

## Supporting Information Available

Kinetics of monomer loss at a range of concentrations; BCM analysis of Fab thermal stability; relative aggregation rate as affected by viscosity; impact of Tween 80 on kinetics of Fab monomer retention at different concentrations; radius measured by DLS; measurement of protein–protein interaction parameter *k*_D_; Finke–Watzky aggregation kinetic model; and mechanistically derived aggregation models (PDF)
