# Supplementary material for: Enhanced Thermal Stability and Reduced Aggregation in an Antibody Fab Fragment at Elevated Concentrations
Source: Mol Pharm. 2023 Apr 11;20(5):2650–61. doi: 10.1021/acs.molpharmaceut.3c00081 (PMC10155210; doi:10.1021/acs.molpharmaceut.3c00081)
Supplement: Supplementary file 1 — mp3c00081_si_001.pdf [file mp3c00081_si_001.pdf]

SUPPLEMENTARY INFORMATION

Enhanced thermal stability and reduced aggregation in an antibody Fab fragment at elevated concentration

Cheng Zhang†, Jordan W. Bye§, Lok H. Lui‡, Hongyu Zhang†, John Hales†, Steve Brocchini‡, Robin A. Curtis§, Paul A. Dalby*, †

†Department of Biochemical Engineering, University College London, Gordon Street, London, WC1E 7JE, UK

§School of Chemical Engineering and Analytical Science, The University of Manchester, Sackville Street, Manchester M13 9PL, U.K

‡UCL School of Pharmacy, 29-39 Brunswick Square, London, WC1N 1AX, UK

*Correspondence email: p.dalby@ucl.ac.uk

Key words: Fab, aggregation, melting temperature (T_m), entropy change (ΔS_{vh}), crowding effect, concentration

1 Kinetics of Monomer Loss at a Range of Concentrations

The observed initial (time = 0) aggregation rates for Fab at different concentrations are shown in

Table S1 based on molecular weight 47378.1 g/mol by supplying its sequence to https://web.expasy.org/compute_pi/.

Table S1. The initial observed aggregation kinetic rate constants and rates for Fab

c (mg/ml)	c (M)	k_{obs} (min^{-1})	v_{obs} ($M\ s^{-1}$)
0.05	1.06E-06	3.95E-02	6.94E-10
0.1	2.11E-06	4.31E-02	1.52E-09
0.2	4.22E-06	4.17E-02	2.93E-09
0.4	8.44E-06	4.15E-02	5.84E-09
1	2.11E-05	4.11E-02	1.45E-08
2	4.22E-05	3.53E-02	2.48E-08
4	8.44E-05	2.69E-02	3.78E-08
8	1.69E-04	2.11E-02	5.95E-08
25	5.28E-04	5.97E-03	5.25E-08
50	1.06E-03	1.78E-03	3.13E-08
100	2.11E-03	5.78E-04	2.03E-08

Table S2. The observed aggregation rate order at different protein concentrations.

MW	concentration			ln(v)		**Rate Order**
(g/mol)	($mg\ mL^{-1}$)	($mol\ L^{-1}$)	ln(conc) ($mol\ L^{-1}$)	(% day^{-1})	($mol\ L^{-1}\ h^{-1}$)	$\frac{d(\ln(v))}{d(\ln(conc))}$
47394.16	0.05	1.05E-06	-13.762	8.494	-13.051	**0.936**
47394.16	0.1	2.11E-06	-13.069	8.618	-12.234	**1.024**
47394.16	0.2	4.22E-06	-12.376	8.617	-11.542	**1.056**
47394.16	0.4	8.44E-06	-11.683	8.628	-10.838	**1.031**
47394.16	1	2.11E-05	-10.766	8.630	-9.920	**0.911**
47394.16	2	4.22E-05	-10.073	8.495	-9.361	**0.754**
47394.16	4	8.44E-05	-9.380	8.245	-8.918	**0.540**
47394.16	8	1.69E-04	-8.687	7.992	-8.478	**0.270**
47394.16	25	5.27E-04	-7.547	6.694	-8.637	**-0.298**
47394.16	50	0.00105	-6.854	5.466	-9.171	**-0.719**
47394.16	100	0.00211	-6.161	4.420	-9.524	**-1.197**

2 Barycentric mean (BCM) analysis of Fab thermal stability

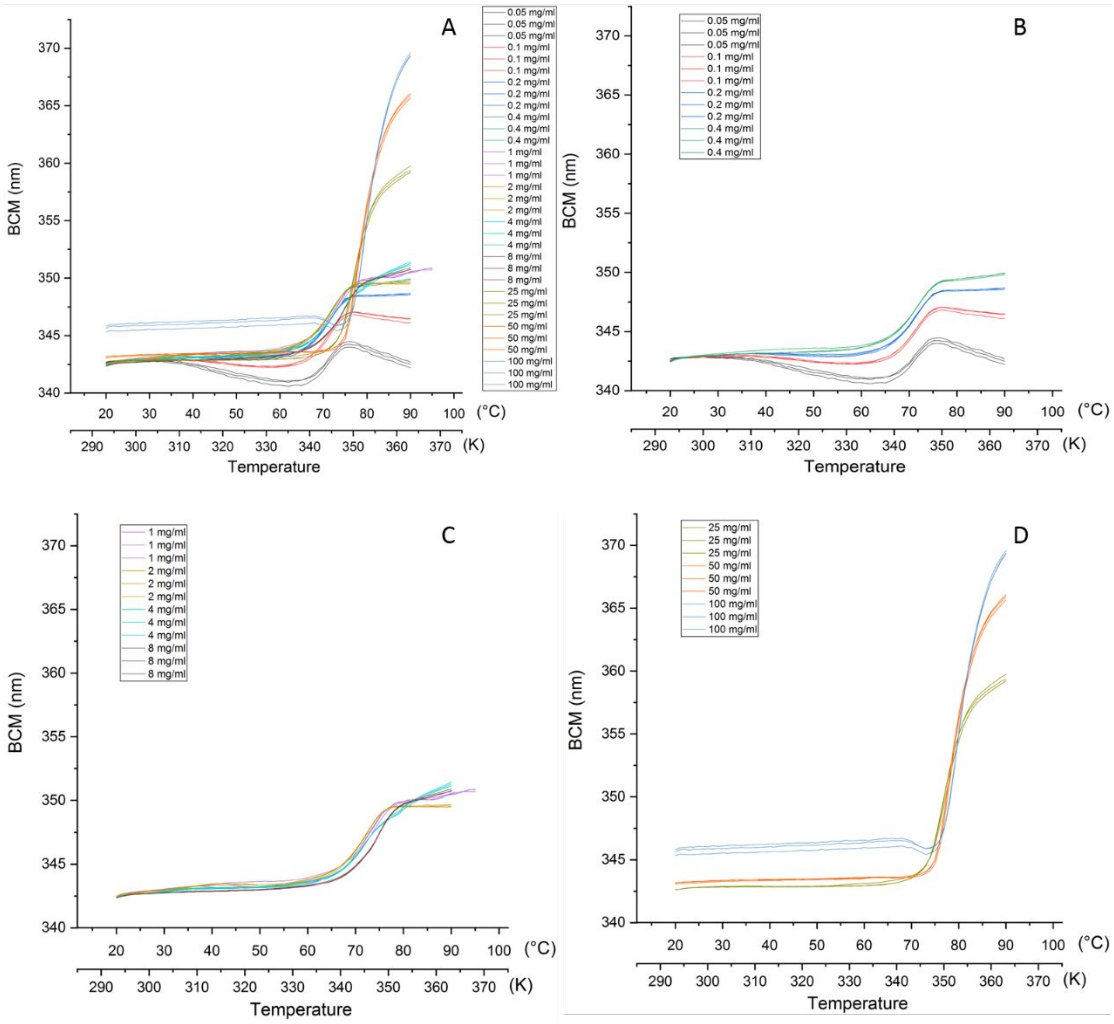

Figure S1. The barycentric mean (BCM) of Fab for the thermal stability analysis. The Fab samples were subjected to a thermal heating treatment from 20 to 90 °C at concentrations from 0.05 to 100 mg/ml in triplicates. *Figure* (A) The BCM data of all the concentrations were plotted together; *Figure* (B) The BCM data of concentrations from 0.05 to 0.4 mg/ml; *Figure* (C) The BCM data of concentrations from 1 to 8 mg/ml; *Figure* (D) The BCM data of concentrations from 25 to 100 mg/ml.

3 Relative aggregation rate as affected by viscosity

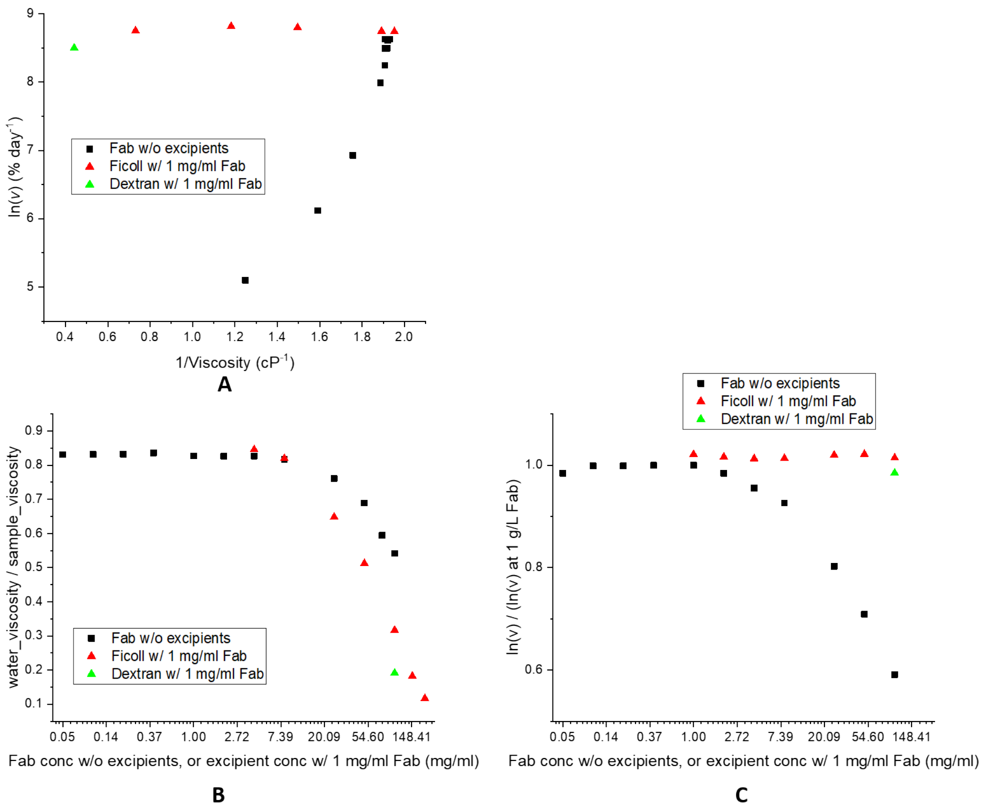

Figure S2. The relative aggregation rate (ln(v), (% day^{-1})) as affected by the viscosity. Figures are re-constructed based on Figure 3 AB. (A) The aggregation rate is plotted against the reciprocal of viscosity; (B) Derived from Figure 3A, where the viscosity is normalised by the water viscosity[11] at 65°C; (C) Derived from Figure 3B, where the aggregate rate is normalised by the rate of 1 g/L Fab without excipients.

4 Impact of Tween 80 on kinetics of Fab monomer retention at different concentrations

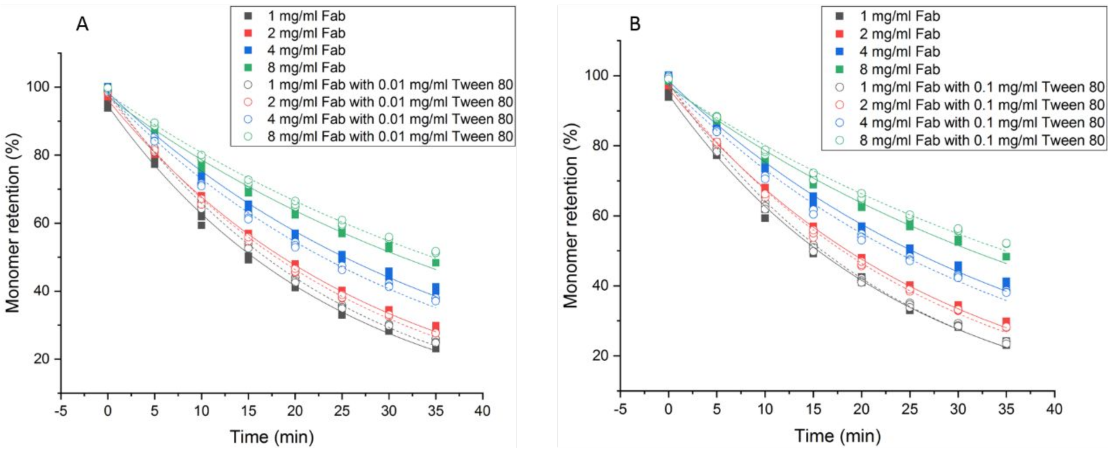

Figure S3. Comparison of Fab monomer retention kinetics with or without the addition of Tween 80, for Fab concentrations at 1 to 8 mg/ml. Samples were incubated at 65 °C and sacrificed in triplicates every 5 min, and was analysed by SEC-HPLC. An exponential function was used to fit each kinetics of monomer retention, with the initial relative aggregate rate shown in the main manuscript.

5 Radius measured by DLS

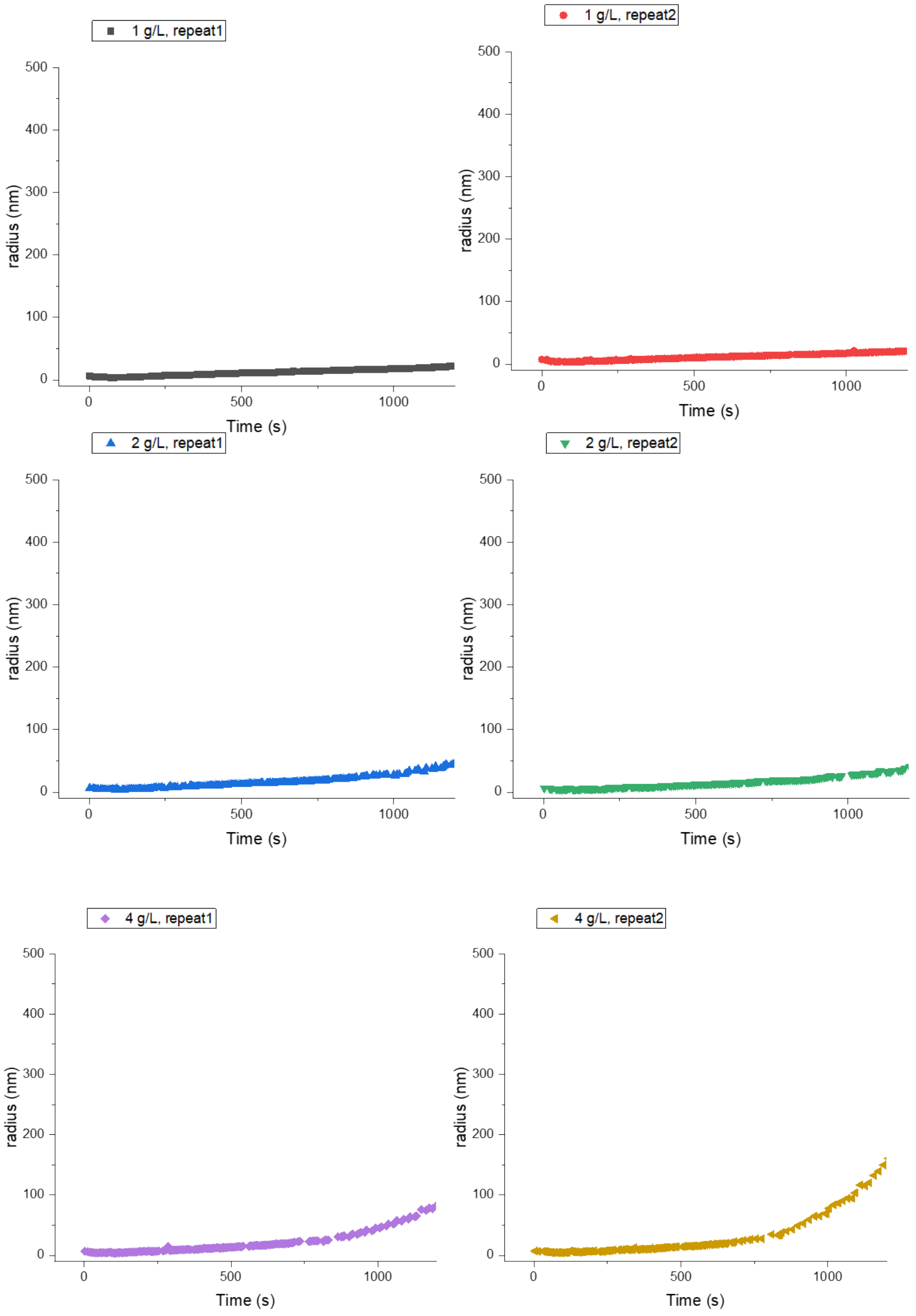

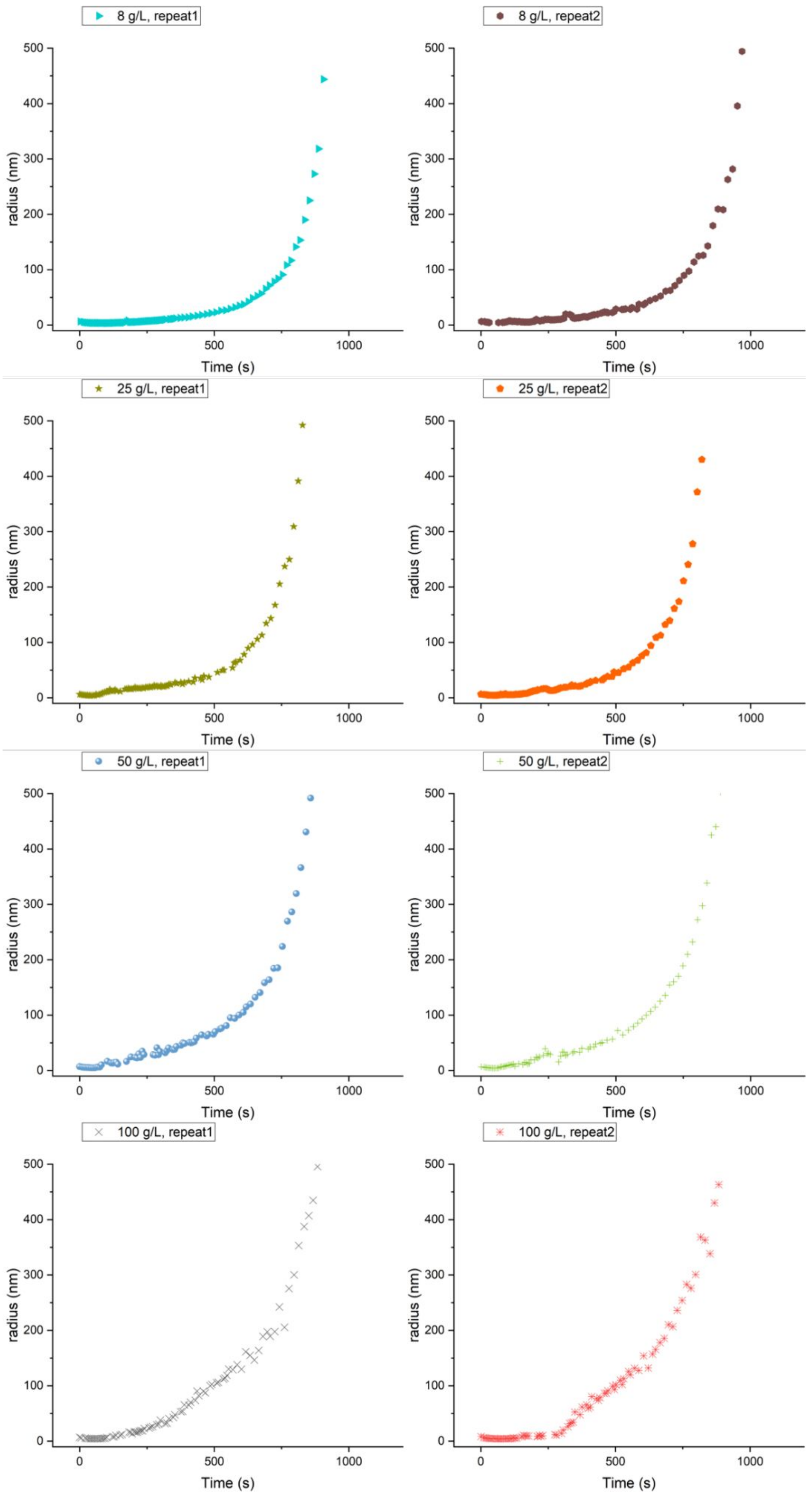

Figure S4. Change in average hydrodynamic radius over time, as measured by DLS at each Fab concentration.

6 Measurement of protein-protein interaction parameter

k_D as a function of temperature

The protein-protein interaction parameter k_D was derived from DLS measurement on a DynaPro Plate Reader (Wyatt Technology, UK). Eleven concentrations from 2.8 to 82 mg/ml were filled at 30 µL/well in triplicates into a black 384-well plate, followed by centrifugation to remove bubbles. One drop of silicone oil was added to each well to avoid evaporation during heating. DLS measurement was performed for all the 33 samples (one-sequence measurement) as samples were linearly heated at 5 °C per sequence between 25-45 °C and 1 °C per sequence between 45-60 °C. The measured hydrodynamic radius (R_h) were plotted against protein concentrations, and was fitted to a linear regression with intercept as the R_{h0} at infinite dilution. R_{h0}/R_h were plotted against protein concentrations, and was fitted to a linear regression with the slope as k_D.

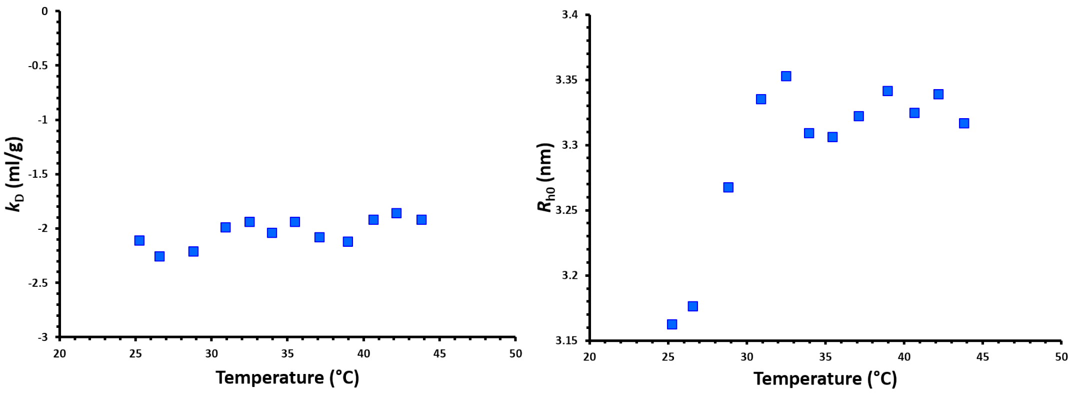

Figure S5. The protein-protein interaction parameter k_D and protein radius at diluted concentration R_{h0}. For each temperature, the hydrodynamic radii R_h measured by the DLS were plotted against a series of protein concentrations, with R_{h0} obtained from the intercept of linear curve fitting. Then for each temperature, the R_{h0}/R_h were plotted against the protein concentration, with k_D obtained from the slope of linear curve fitting.

7 Protein-protein interactions at room temperature

Figure S6 below shows the long-wavelength limit of the structure factor *S0* and the mutual diffusion coefficient measured using the Nanostar on samples with protein concentrations ranging from 1 to 100 g/L. *S0* was obtained from the light scattering equation

$$S0 = \frac{1}{M_{\mathrm{w}}Kc} R_{\theta}$$

where R_{θ} is the excess Rayleigh ratio, *K* is a light scattering constant, and M_{w} is the weight average protein molecular weight obtained from extrapolating the low concentration data. *S0* is related to the Kirkwood-Buff protein-protein interaction parameter by $S0 = 1 + c_2 G_{22}$. The measured molecular weight M_{w}obtained by extrapolating the low protein concentration data was equal to 51.4 kDa, which was slightly greater than the sequence molecular weight equal to 47.4 kDa.

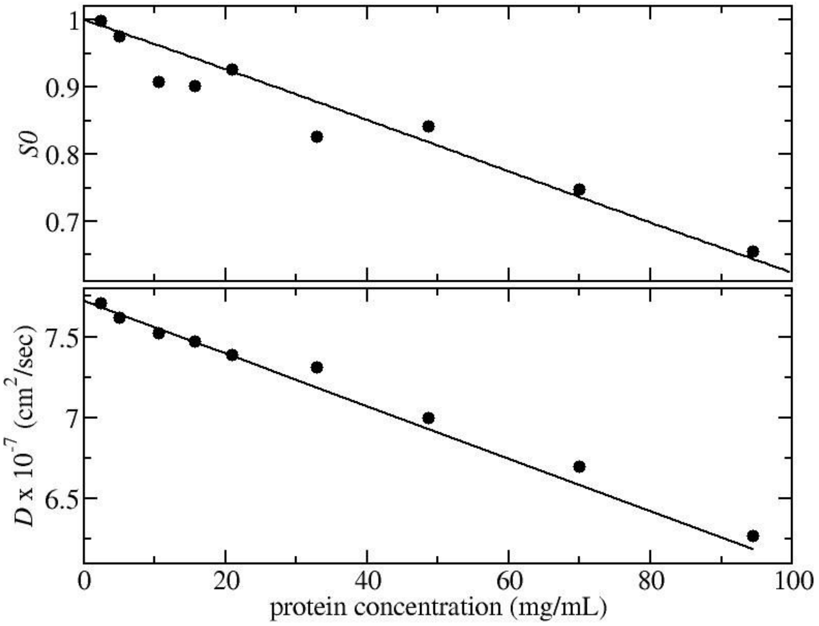

Figure S6. The top graph shows the long-wavelength limit of the structure factor *S0* obtained by SLS and the line is the best fit to the Baxter model over the full protein concentration range. The lower graph shows the measured diffusion coefficient obtained by DLS as a function of protein concentration and the best fit line to the data at a protein concentration less than 20 mg/mL.

The SLS and DLS measurements were used to check the levels of protein-protein association under native conditions. Under moderate ionic strength conditions greater than 200 mM, electrostatic repulsion is screened sufficiently that thermodynamic properties for solutions of weakly interacting proteins can be described using an adhesive hard sphere (Baxter) model, where protein-protein interactions include a steric repulsion and a spherically-symmetric sticky potential.[14] *S0*, which is equal to the inverse of the osmotic compressibility, has been fit to the model over the full protein concentration range including the effects of multibody interactions. The only fit parameter in the model is the stickiness parameter τ, where the hard sphere diameter was set equal to the measured hydrodynamic diameter by DLS. The fit value of $\tau = 0.36$ corresponds to a reduced osmotic second virial coefficient of $b_{22} = 0.30$, which provides an indication that there are weakly attractive protein-protein interactions. The k_D value obtained from fitting to the diffusion coefficient data is equal to -2.1 mL/g and also reflects a similar level of weakly attractive protein-protein interactions. This can be confirmed by comparing the experimentally-derived value to the value predicted by the Baxter model using $\tau = 0.36$, which gives $k_\mathrm{D} = -1.7$ mL/g.[15] Taken together, the results do indicate that protein-protein interactions are weakly attractive up to high protein concentrations, but there is no evidence of strong reversible self-association since the *S0* profile can be captured using a spherically symmetric interaction potential.

8 The Finke-Watzky aggregation kinetic model

8.1 Methods

Non-native protein aggregation has been extensively studied with various mechanisms and kinetic models[1,2]. Elucidating the rate-limiting steps would offer insight into potential molecular mechanisms that could explain the observed behaviour.

The Finke-Watzky (FW) model[4] averages and simplifies the thousands of elementary steps potentially involved in actual protein aggregation. However, it has adequately

captured the aggregation profiles for many datasets related to prion and neurodegenerative disorders[1], full-length monoclonal antibody (mAb)[5–7], antibody light chain[8] and immunoglobulin-like domains[9]. However, it has not been applied previously to study the aggregation of a Fab.

The Finke-Watzky (FW) model assumes a slow continuous formation of nucleation sites with rate constant k_1; and then the autocatalytic surface growth (i.e. irreversible elongation) of new nuclei through monomer addition to existing nuclei, with rate constant k_2, which accelerates aggregate growth (SI_Scheme 1). The derivative equation (SI_Equation 1) and its integrated form (SI_Equation 2) indicate that the two rate constants can be obtained by fitting a series of time-dependant concentration throughout the aggregation. The initial concentration of the protein $[A]_0$ was set to 100% (SI_Equation 3) and fitted to the monomer retention (Figure 1A in MS) using OriginPro 2019 Software (9.6.0). Therefore, the unit for k_1 and k_2 are both min^{-1}.

$$A \xrightarrow{k_1} B$$

$$A + B \xrightarrow{k_2} 2B \qquad \text{SI\_Scheme 1}$$

$$\frac{-\mathrm{d}[A]}{\mathrm{d}t} = k_1[A] + k_2[A][B] \qquad \text{SI\_Equation 1}$$

$$[A]_t = \frac{\frac{k_1}{k_2} + [A]_0}{1 + \frac{k_1}{k_2[A]_0}\exp\,(k_1 + k_2[A]_0)t} \qquad \text{SI\_Equation 2}$$

$$[A]_t = \frac{\frac{k_1}{k_2} + 1}{1 + \frac{k_1}{k_2}\exp\,(k_1 + k_2)t} \qquad \text{SI\_Equation 3}$$

8.2 Results

A typical kinetic curve for the F-W model exhibits a sigmoidal character with a lag phase, i.e. an induction period, but a curve without evident lag phase could still be well fitted[4]. In our case, the lag phase is not evident. The rate constants (Figure S7C-E) show

that k_2 >> k_1 at all concentrations, implying that the rate of elongation through monomer addition to existing aggregates, was far more rapid than the conversion from native monomers to the aggregate nuclei. Therefore, the nucleation is the rate-limiting step. figure S7C shows that the nucleation rate remains stable around 0.005-0.023 min^{-1} below 8 mg/ml, mildly decreased to 3.8 x 10^{-4} min^{-1} at 25 mg/ml, followed by a substantial drop to 3.6-6.1 x 10^{-7} min^{-1} at 50-100 mg/ml. This trend corresponds to the rate order (Figure 1C in MS), in which the aggregation rate witnesses a relative slow decrease from 0.05 to 8 mg/ml, followed by a considerable reduction above 25 mg/ml. As the nucleation rate is presumably the rate-limiting step, it is not surprising that the aggregation rate is dominated by the first nucleation step of the FW model. The rate of aggregate growth (Figure S7D) was comparable to each other across all the concentration, though a steady increase was seen at higher concentration.

It should be noted that the FW model alone could not explain the protection effect at increasing protein concentrations, and instead results in concentration-dependent rate constants with no mechanistic basis.

Next, we investigated models with global fitting strategies[3] that assume that all protein concentrations share the same set of kinetic parameters.

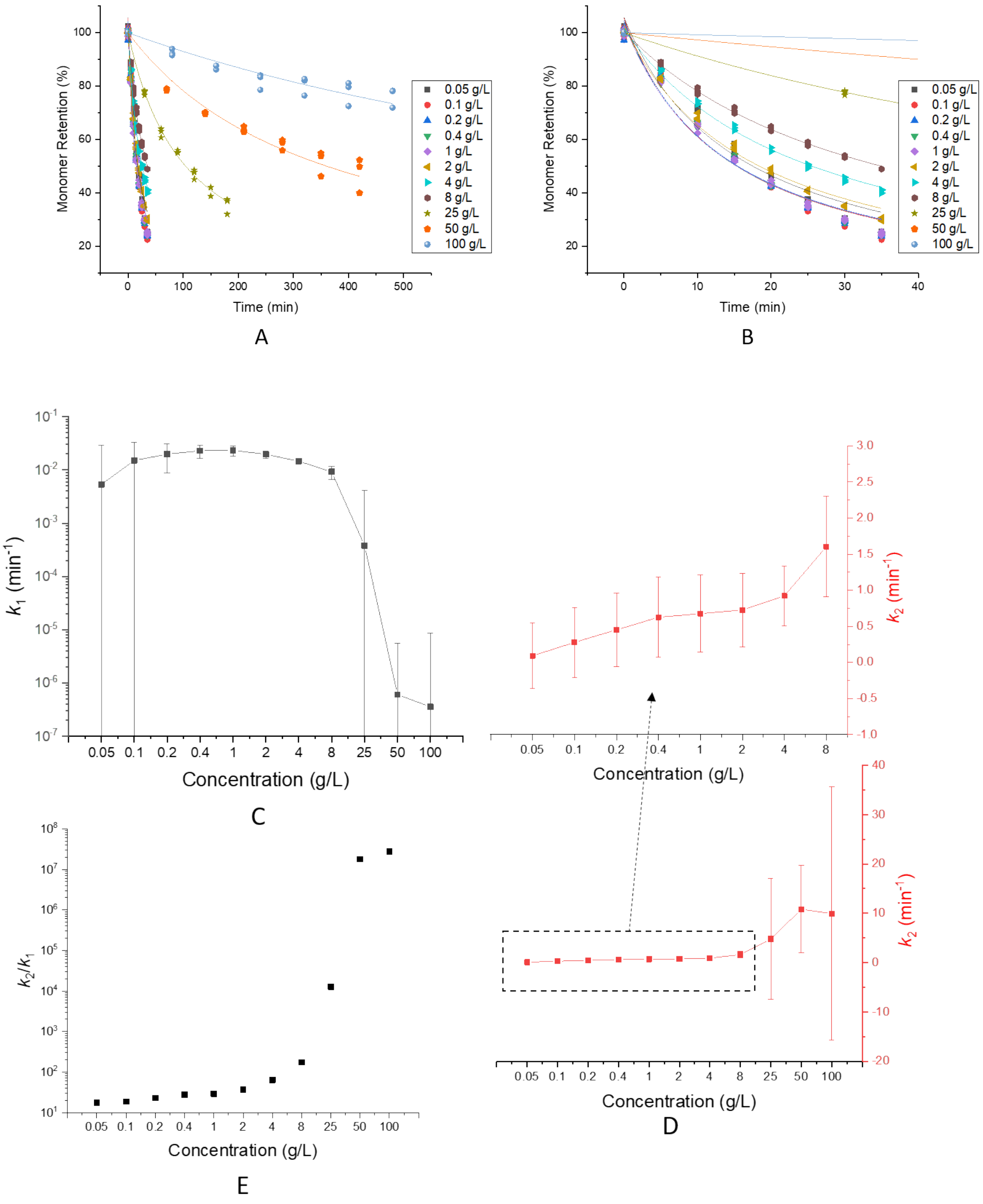

Figure S7. The Finke-Watzky model fitting to the Fab monomer retention. The monomer retention (Figure 1A in MS) was fitted to the F-W model up to 500 min (A) and the first 35 min (B). The fitted kinetic rate constants k_1 and k_2 are shown in (C) and (D) for nucleation and aggregate growth, respectively, with standard error as the error bars. The ratio of k_2/k_1 is shown in (E).

9 Mechanistically-derived aggregation models

9.1 Justification for more complex models

If in the simplest case we assume that the aggregation is triggered solely by *free* monomers (c'), and with a single fixed rate constant k_1 (e.g. 4.45E-02 s^{-1}), then $v_{obs} = k_1$*.c'. In this case to fit the observed rate data, the fraction of monomeric protein c'/c at each total protein concentration, must decrease either exponentially or hyperbolically (Figure S8). Therefore, it is clear that for an aggregation mechanism that assumes a fixed rate constant for conversion of monomer to aggregate, then a mechanistic feature needs to be introduced that alters either c'/c, or the reaction rate order, such that there is less oligomerisation from a native-like conformer at increased concentration.

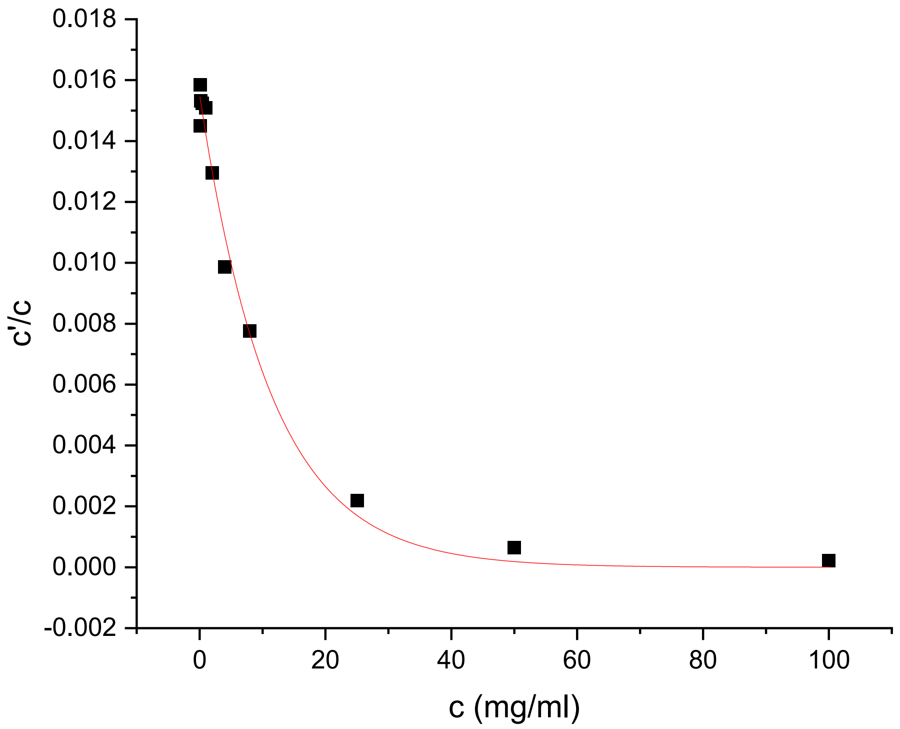

Figure S8. The ratio of free monomer over total monomer. The data is fitted to an exponetial equation with R^2 = 0.99.

In fact, the rate order changes from approximately 1 (0.05-1 mg/ml) to -1.1 (100 mg/ml) (Figure S9). However, a negative rate order for a single reaction step is not possible and should be attributed to mixed pathways or multiple mechanisms that affected the kinetic constant(s) at increased concentration. In the discussion below, various kinetic models are assessed to determine possible best fits to the observed kinetic data.

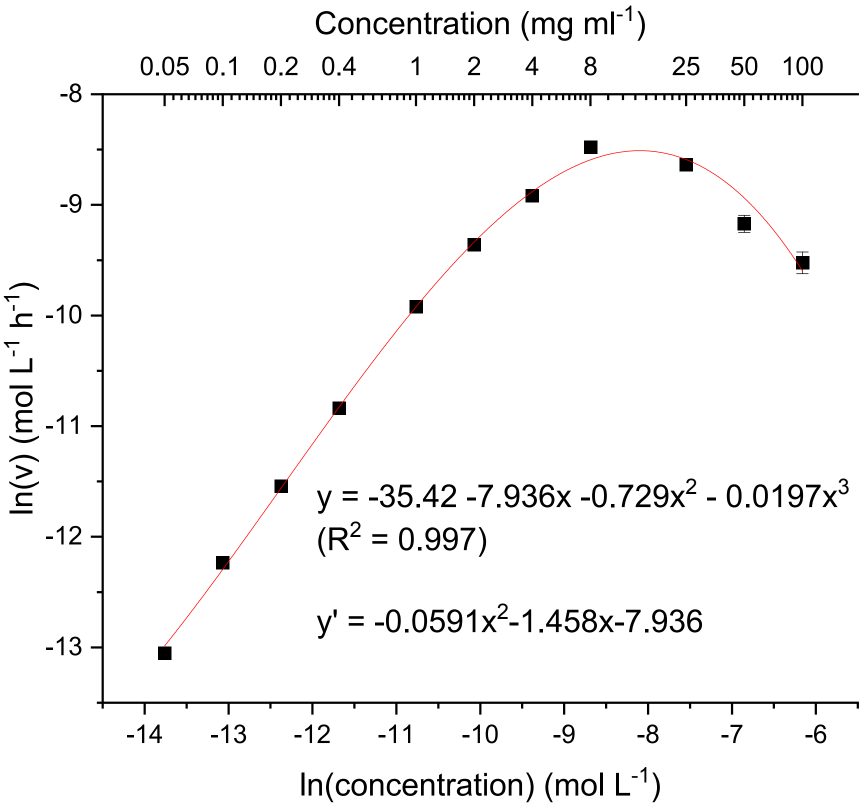

Figure S9. The change of the rate order at different concentrations. A third-order polynomial equation was used to fit the data. The slope at each concentration corresponds to the rate order and is tabulated in

Table S2.

9.2 Variable reaction order model

Fitting a single reaction rate order, m, that is invariable to protein concentration, does not fit the observed data for any value of m. Values of m<1 lead to hyperbolic dependencies on protein concentration, with a gradual approach to a maximum rate at high concentrations. Values of m>1 lead to rapid increases in reaction rate with protein

concentration. A gradual change in the reaction rate order for aggregation from monomers could lead to slower reactions at higher concentrations, whereby the requirement for more monomers to come together in each successful forward reaction, also increases. This can be simply modelled as:

$v_{obs} = k_1^{*}.c' = k_1^{*}.c^{m}$.

where a scaling function m = 1+m'*(c/c0), essentially forced the initial rate order to be 1, and then scaled with concentration c, as a function of the initial concentration c0.

The model converged reasonably well (R^2 = 0.94), with m'=0.00045 such that m ranged from 1 to 1.9. This simulated the overall profile, but it was also clear that it failed to account for the levelling off of the rate at higher concentrations, where the model instead descended asymptotically towards zero. More complex modifications of the scaling function for m could no doubt be found that fit the data perfectly well. Despite fitting well, the mechanistic basis for a varying rate order was not defined. A switch to the second order (m=1.9) reaction must occur along with a gradual *exclusion* of the first order reaction. This would imply a gradual shift in monomer conformation, or free monomer availability, with increasing protein concentration, whereby the original monomer at low concentration has m=1, and the new conformation has m=1.9. Monomer self-crowding or oligomerisation effects could potentially provide such a mechanism.

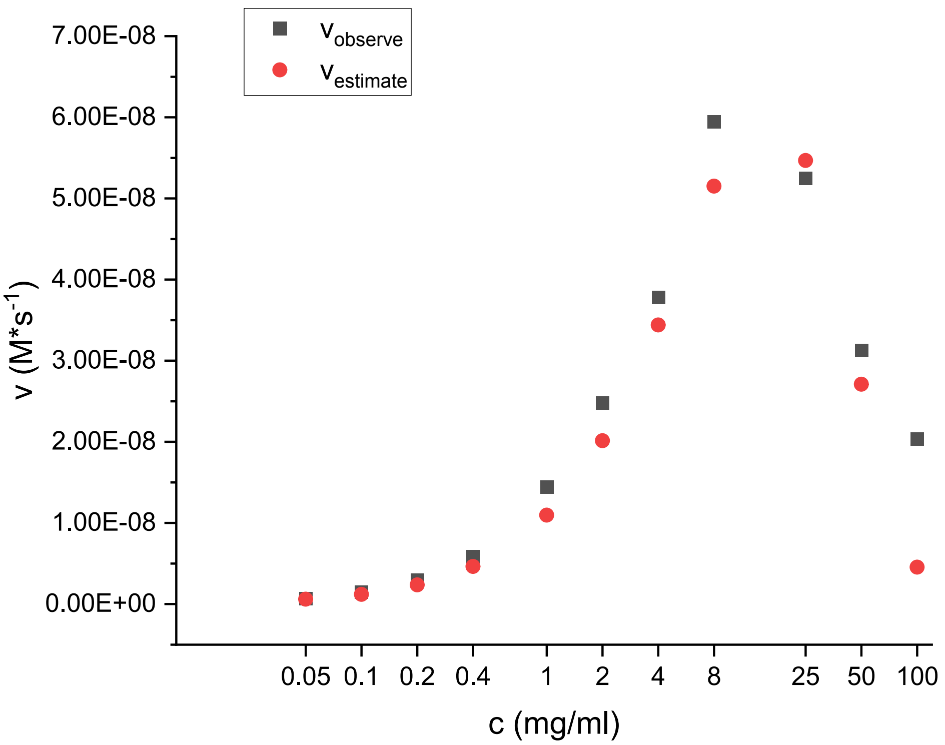

Figure S10. Fit to the variable reaction order model.

9.3 Two-state monomer conformation switch

Mathematically, for a shift in conformation only, for example due to macromolecular self-crowding, we could assume a 2-state equilibrium shift from low concentration monomer population c' to higher concentration monomer population c''. This could reflect for example an open conformation of the monomer, c', that is aggregation prone (N*), and a closed form, c'', that is not aggregation prone (N). By analogy with chemical or thermal denaturation transitions, the c' proportion in the total c is expressed as

$$\frac{c'}{c} = \frac{\mathrm{D} + \mathrm{b}.c + \ c_0.e^{m(c - c_{50})}}{1 + e^{m.(c - c_{50})}}$$

where c_0 is the maximum concentration of N* found under low total concentration conditions. We fixed this simply as the lowest concentration of protein added, assuming that essentially all of the protein is N*. D is the lowest concentration of N* and is found

under high total concentration conditions. b is an underlying baseline slope for N* vs c in the high concentration region. *m* is the cooperativity of the transition.

Only the c’ monomer form was assigned for aggregate formation with rate order m’, where:

$$v_{est} = k_1.c'^{m'} = k_1.(c.\frac{c'}{c})^{m'}$$

Attempts to solve the model, allowing D, b, m, c_{50}, m', k_1 to vary, converged well with R^2 = 0.997. It was found that R^2 could be maximised from only five parameters, without varying k_1, but then k_1 could be optimised afterwards to ensure that $v_{observe}$ = $v_{estimate}$, through converging towards a slope = 1 for $v_{observe}$ vs $v_{estimate}$. The model led to a decrease in the observed rate with increasing protein concentration. Thus a 2-state conformational equilibrium was a good proxy for macromolecular self-crowding effects where they are expected to shift the population of monomers from aggregation-prone c' to non-aggregation-prone c''.

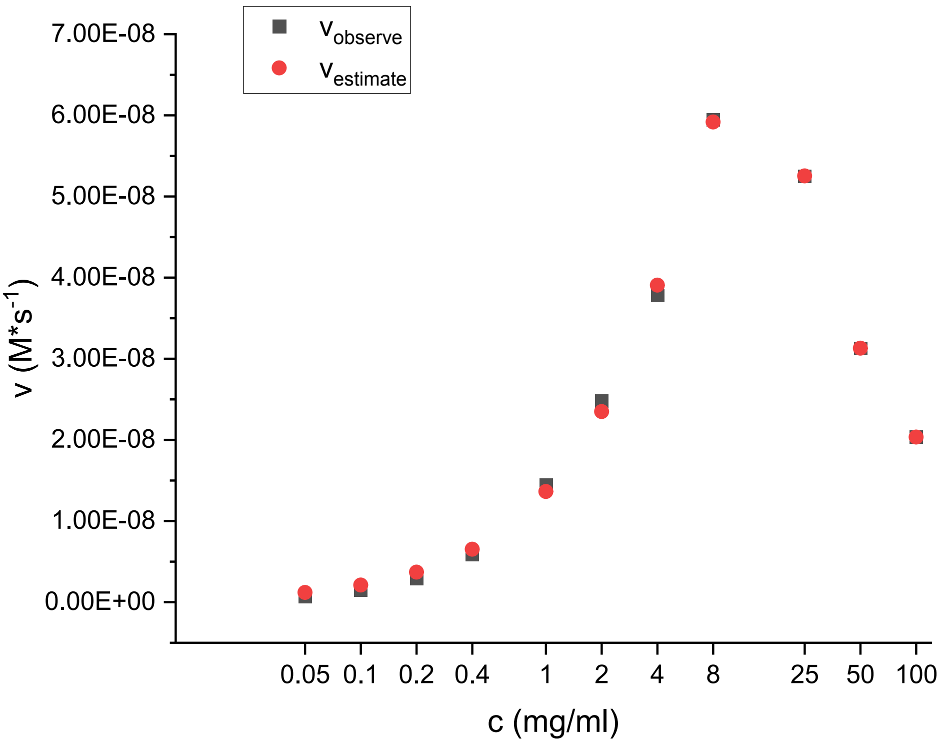

Figure S11. Fit to a model based on two-state monomer conformation switch.

9.4 Off pathway oligomer models

An off-pathway dimer or other oligomer that does not itself aggregate could also potentially lead to slower aggregation at higher protein concentrations by removing the availability of free monomer. Here, the free monomers are assumed to initially equilibrate to an oligomer (expressed in the dissociation direction): $c_n \overset{K}{\Leftrightarrow} n.c'$

where c' is the free monomer concentration in solution after rapid pre-equilibration with monomers self-associated into c_n, with equilibrium constant K.

Thus, $K = \frac{c'^n}{c_n}$

Because $c = c' + n.c_n,\ \ c_n = \frac{c - c'}{n}$

Thus, $K = \frac{c'^n.n}{c - c'}$, or $n.c'^n + K.c' - K.c\ = 0$

If $n = 2$, $2.c'^2 + K.c' - K.c\ = 0$, thus $c' = \frac{-K\ + \sqrt{K^2 + 8Kc}}{4}$ (only keep the positive solution)

The c’ will always be assumed based on formation of a dimer in the models discussed below, if not explicitly explained.

9.5 Monomolecular aggregation with off pathway dimer D

In this model, the estimated rate was modelled as v_{est} = c’.k, assuming a monomolecular reaction to form aggregate from the free monomer. A monomolecular reaction appears to be justified by the rate order of 1 at lower protein concentrations.

The resulting fitted parameters do not converge with a good fit to the observed rates, as the shape of the equation being fitted does not match the concentration profile of the observed data.

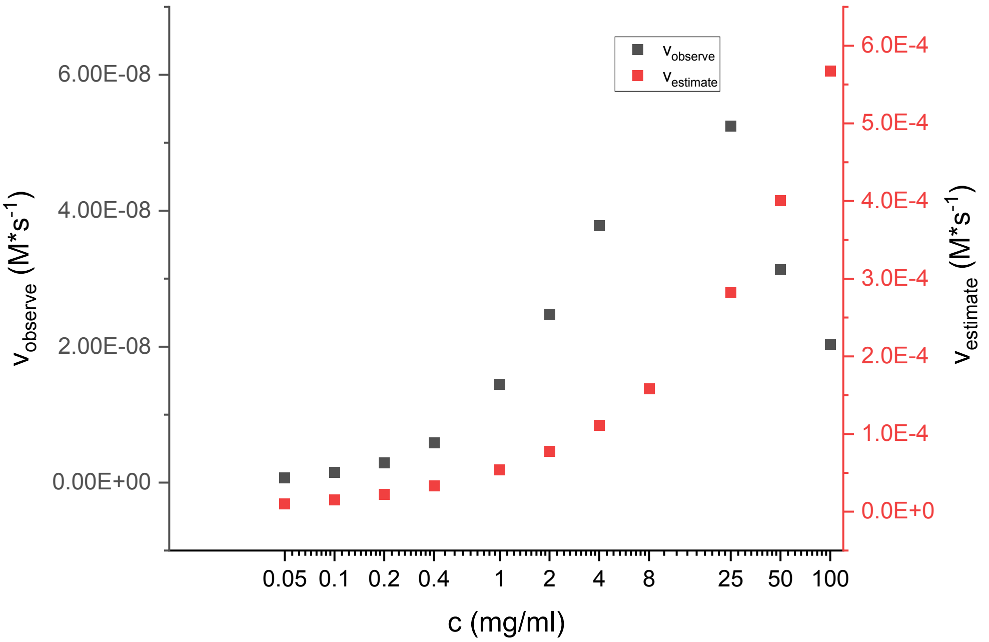

Figure S12. Fit to a model of monomolecular aggregation with off pathway dimer D.

9.6 Bi-molecular aggregation with off pathway dimer D

In this model, the estimated rate was modelled as $v_{est} = c'^2.k$, to assume a bi-molecular reaction to form aggregate from the free monomers.

The resulting fitted parameters do not converge with a good fit to the observed rates, as the shape of the equation being fitted does not match the concentration profile of the observed data.

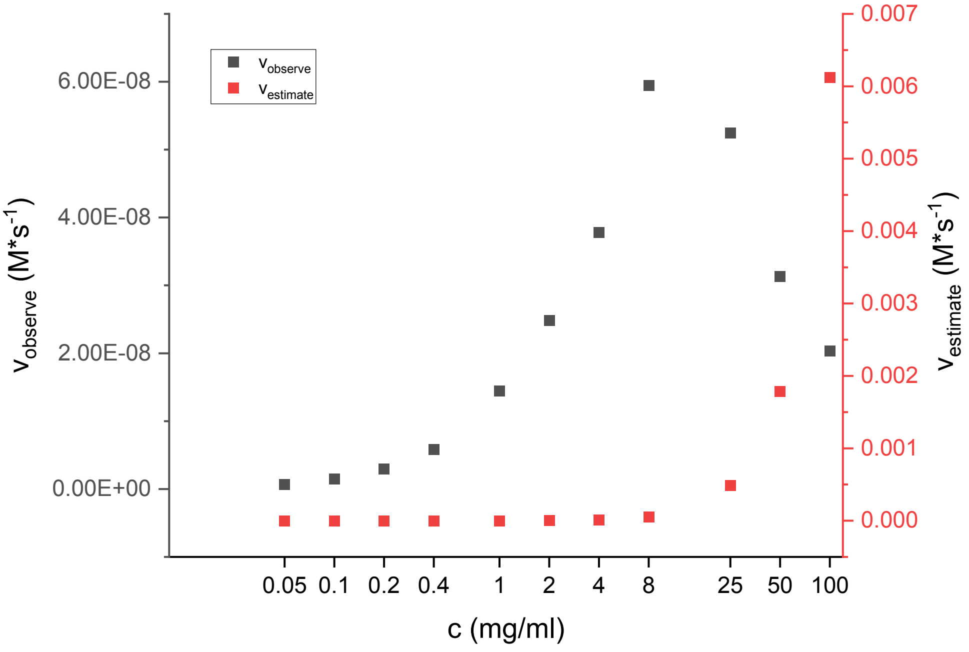

Figure S13. Fit to a model of bi-molecular aggregation with off pathway dimer D.

9.7 Undefined aggregation rate order, with off pathway dimer D

In this model, the estimated rate was modelled as $v_{est} = c'^{m}.k$ to allow the model to fit for any single reaction rate-order to form aggregate from free monomers.

The resulting fitted parameters did not converge with a good fit to the observed rates, as the shape of the equation being fitted did not match the concentration profile of the observed data.

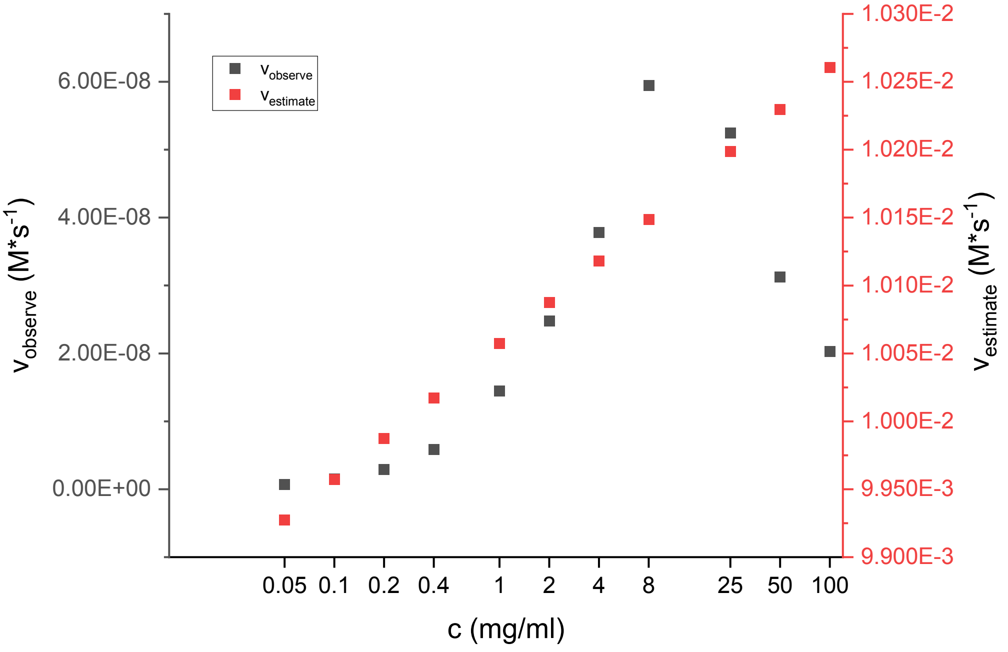

Figure S14. Fit to a model with undefined aggregation rate order, and off-pathway dimer.

9.8 Modelled $v = k_m.c'^m - k_d.D^d.c'^{(c)}$ with off pathway dimer D

In this model, the estimated rate was modelled as $v_{est} = k_m.c'^m - k_d.D^d.c'^{(c)}$. The dimer presence inhibits the aggregate formation by kinetically associating with free monomer, proportional to $D^d.c'^{(c)}$.

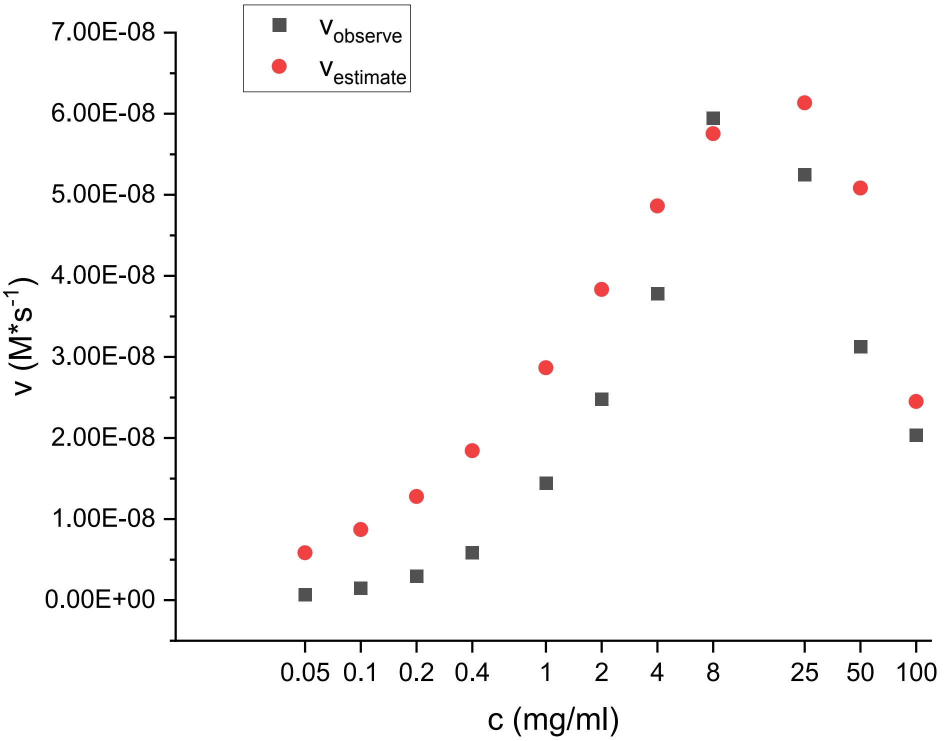

Figure S15. Fit to a model based on $v = k_m.c'^m - k_d.D^d.c'^{(c)}$ with off pathway dimer.

The model was overparameterised. The final model gave an RSQ 0.92.

9.9 Modelled for an off pathway dimer D as an inhibitor.

In this model, the estimated rate was modelled as $v_{est} = k_n/(1+D/K_i).c'$. The dimer presence inhibits the aggregate formation by associating with free monomer at equilibrium, modifying the rate constant k_n to $k_n/(1+D/K_i)$.

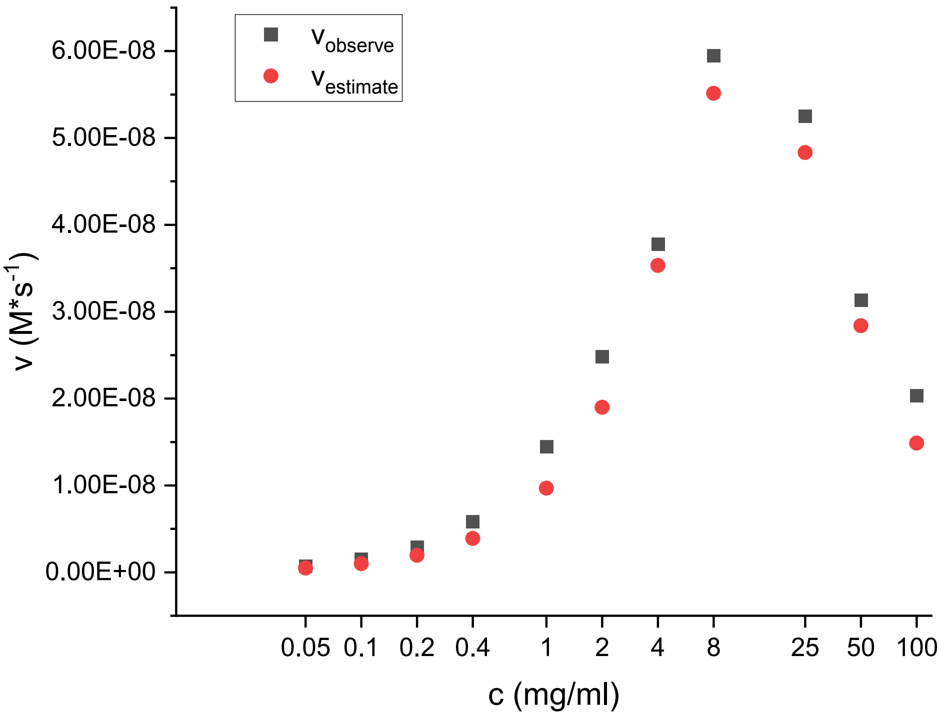

Figure S16. Fit to a model for an off pathway dimer D as an inhibitor.

This model converged well with good fit. The final model gave an $R^2 = 0.99$, $K = 1.72$ M, $k_n = 4.61\text{E-}04\ s^{-1}$, $K_i = 4.01\text{E-}08$ M.

9.10 Model summary

Table S3. The potential mechanistic models for the Fab aggregation reactions

Model	Equation	Schematic Diagram	Parameter Values
1. Unimolecular from free monomer c'. No dimer.	v_{est} = c'.k	M → Agg	N/A
2. Variable reaction order model	v_{est} = k.c^m<br>m = 1+m'.(c/c0)<br>$v_{est} = \mathrm{k}.c^{1+\mathrm{m'}.(\mathrm{c}/\mathrm{c}0)}$	M → Agg	**R^2 = 0.94**<br>k = 0.000572 s^{-1} M^{1-m}<br>m' = 0.00045
3. Two-state monomer conformation switch model	$v_{est} = k_1.c'^{m'} = k_1.(c.\frac{c'}{c})^{m'}$<br>$\frac{c'}{c} = \frac{D + b.c + c_0.e^{m(c-c_{50})}}{1+e^{m.(c-c_{50})}}$	M → M'<br>M' → Agg<br>M' → M''	**R^2 = 0.997**<br>m' = 0.82<br>c_{50} = 1.98x10^{-4} M<br>D = 6.61x10^{-8} M<br>k_1 = 9.336 $M^{1-m'}s^{-1}$<br>b = -2.51x10^{-5}<br>m = -6226 M^{-1}
4. Unimolecular with off pathway dimer D	v_{est} = c'.k<br>$c' = \frac{-K + \sqrt{K^2+8Kc}}{4}$	M ⇌ D<br>M → Agg	N/A<br>varied k, K
5. Bimolecular with off pathway dimer D	v_{est} = c'^2.k<br>$c' = \frac{-K + \sqrt{K^2+8Kc}}{4}$	M ⇌ D<br>2 M → Agg	N/A<br>varied k, K
6. Variable rate-order with off pathway dimer D	v_{est} = c'^m.k<br>$c' = \frac{-K + \sqrt{K^2+8Kc}}{4}$	M ⇌ D<br>m.M → Agg	N/A<br>varied k, K, m
7. Model with off pathway dimer D	v = k_m.c'^m - k_d.D^d.$c'^{(c)}$<br>$c' = \frac{-K + \sqrt{K^2+8Kc}}{4}$	M ⇌ D<br>(c).M + d.D → Oligo<br>m.M → Agg	**R^2 = 0.92**<br>k_m = 0.000359<br>m = 0.71

and higher-order oligomers	D = (c-c')/2		k_d = 7.9 x 10^{-5}<br>d = 0.26<br>K = 0.00078 M
8. Model with off pathway dimer D as an inhibitor	v = $k_n/(1+D/K_i).c'$<br>$c' = \frac{-K + \sqrt{K^2 + 8Kc}}{4}$<br>D = (c-c')/2	M ⇌ D<br>M → Agg (inhibited by D)	**R^2 = 0.99**<br>K = 1.72 M<br>k_n = 4.61E-04 s^{-1}<br>K_i = 4.01E-08 M

Overall, a macromolecular crowding effect could be used to explain model 2 (Section 9.2, R^2 = 0.94) and model 3 (Section 9.3, R^2 = 0.997), both of which fit well. In model 2, the rate order of aggregation shifted from 1 to 1.9 as the monomer conformation changed with protein concentration. In model 3, the aggregation was inhibited as monomer shifted from aggregation-prone ones at low concentration to non-aggregation-prone ones at high concentration. Alternatively, an inhibitory dimer in model 8 also fit the data, and with even greater precision (R^2 = 0.99).

9.11 The Lumry-Eyring Nucleated Polymerization(LENP) model[12,13]

We have also interpreted the aggregation data in terms of the Lumry-Eyring nucleated polymerization (LENP) model assuming the folding/unfolding equilibrium is much faster than aggregation rates. This model accounts for a nucleation step involving the formation of critically-sized nuclei and various pathways for aggregate growth. The main purpose of using this model was to provide insight into the relative rates of aggregate growth with respect to aggregate formation from considering the monomer loss kinetics as well as the simultaneous dynamic and static light scattering data obtained from the isothermal Nanostar runs.

The mechanism of aggregate growth can be understood from plotting aggregate molecular weight against the fraction of protein aggregated, 1 - *m*. Because the SLS

signal was saturated for many of the runs, especially at higher protein concentration, we had to estimate the molecular weight by assuming that all aggregates have a self-similar structure in which case the molecular weight can be determined by $M_{\mathrm{w}} \propto R_{\mathrm{H}}^{\mathrm{d}_f}$. d_{f} is the fractal dimension of the aggregate, which reflects the aggregate structure, where increasing values reflect more compact structures. In Figure S17 below, the simultaneous SLS and DLS data combined with the monomer loss kinetics has been used to estimate d_{f} $= 2.2$ for the runs at 1 g/L. A similar analysis to the data at 2, 4, and 8 g/L has yielded values of d_{f} equal to 1.9, 2.0, and 2.1, respectively. These values fall in the expected range for protein aggregates and provide an indication that the aggregate structure is the same across the runs at different protein concentration. In order to check for the mechanism of aggregate growth, in Figure S17d, we have plotted the values for $R_{\mathrm{H}}^{d_{\mathrm{f}}}$, which is expected to scale with aggregate molecular weight when the contribution from the monomer to the light scattering is negligible, which occurs when $1 - m$ > 0.2. A linear relationship is expected when growth occurs by chain polymerization.[13] Because the fractal dimension of the aggregate is not known precisely, the analysis has been carried out for two different values of d_{f} equal to 1.8 or 2.2. In both cases, there is a clear up-turn in the graph that occurs around $1 - m \approx 0.5$ providing an indication that aggregate-aggregate coalescence is occurring at least at longer times. For shorter times, growth is likely occurring through chain polymerization, although it is not possible to detect when there is a cross-over between the dominant mechanism of aggregate growth.

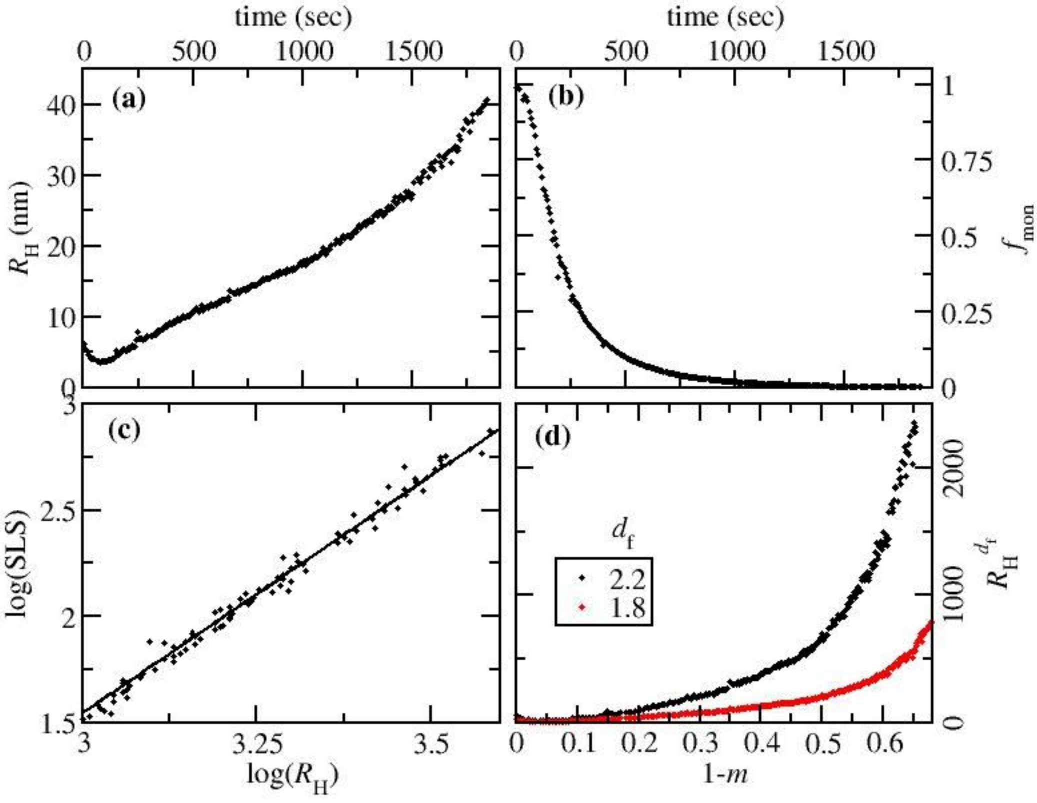

Figure S17: Analysis of the data obtained from the 1 g/L run at 65 °C. In **(a)** there is a plot of the measured R_H as a function of time. In **(b)**, f_mon is the fraction of light scattering by the monomer obtained from the SLS reading and knowledge of the monomer loss kinetics. In **(c)**, the fractal dimension of the aggregate d_f is obtained from plotting $\log{(SLS)}$ versus $\log{(R_\mathrm{H})}$ where the slope of the plot is equal to d_f. The plot is only carried out for the data collected over the range $750 < t(\mathrm{sec}) < 1500$, where the contribution of light scattering by the monomer is negligible and the aggregate size remains less than 30 nm, so there is no angle dependence to the scattered light. This yields a value of $d_\mathrm{f} = 2.2$. In **(d)**, $R_\mathrm{H}^{d_\mathrm{f}}$, which is proportional to aggregate molecular weight, is plotted versus the fraction of protein that is aggregated $1 - m$, where $m = c/c_{t0}$.

In Figure S18, there is a plot of aggregate molecular weight in terms of $R_\mathrm{H}^{d_\mathrm{f}}$ versus the amount of protein that has aggregated. With initial increase in protein concentration, the aggregate size at a fixed amount of aggregated protein decreases up until the run with an initial concentration of 8 mg/mL. Above this concentration, the reverse trend is observed. This behaviour can be rationalized in terms of the relative rates of aggregate growth versus aggregate formation. A faster relative rate causes larger aggregate sizes as a function of the amount of protein in the aggregate. The differences in aggregate growth rate are apparent at very short times perhaps indicating that they arise during growth by chain polymerization.

Within the LENP model, the aggregate formation or nucleation rate scales as $f_R^x c_{t0}^{x-1}$, while the chain polymerization rate is proportional to $f_R^\gamma c_{t0}^\gamma$, where f_R is the fraction of the reactive protein intermediate N*, x is the size of the critical nuclei, and γ, is the size of the growth unit during chain polymerization. The results for the relative rates of aggregate growth versus nucleation, represented by β_gn, shown in Table S4 below, have been estimated for different values of x, with the growth unit fixed to a value equal to 1. We have also assumed that the fraction of the reactive intermediate N*, is proportional to the fraction of unfolded protein, which has been estimated from the unfolding curves. For the case $x = 1$, $\beta_\mathrm{gn} \propto c_\mathrm{t0}$ so that the relative aggregation to nucleation rate is a monotonically increasing function of protein concentration. On the other hand, for $x = 2$, $\beta_\mathrm{gn} \propto f_\mathrm{R}^{-1}$ so that there is a minimum in the relative aggregation rates at 4 g/L, since this concentration corresponds to the least conformationally stable condition with the highest fraction of unfolded protein. With further increasing values of x, the minimum becomes much more pronounced since the nucleation rate has a higher order dependence on f_R. Increasing protein concentration also increases the nucleation rate, but when comparing the run at 4 g/L versus the runs with protein concentrations at 50 or 100 g/L, the controlling factor is the much higher fraction of unfolded protein at 4 g/L.

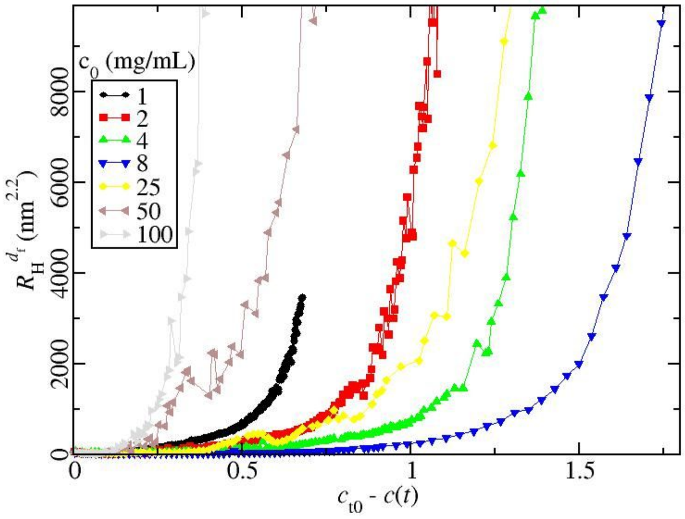

Figure S18: Plot of $R_\mathrm{H}^{d_\mathrm{f}}$ with $d_\mathrm{f} = 2.2$ versus the concentration of aggregated protein determined from combining the monomer loss kinetics with the DLS data.

Table S4: Estimations for the relative rates of aggregate growth by chain polymerization to aggregate nucleation β_{gn} for all the runs relative to the run with $c_{\mathrm{t0}} = 1$ mg/mL.

c_{t0}	$\%f$	$x = 1$	$x = 2$	$x = 3$	$x = 4$
1	3.5	1	1	1	1
2	4.9	1.8	0.71	0.28	0.11
4	8.9	3.1	0.39	0.049	0.006
8	2.0	6.5	1.8	0.47	0.13
25	0.18	20.5	19.6	18.8	18.0
50	0.11	40.3	32.7	26.5	221
100	0.035	79.7	98.0	121	148

10 References

1. Morris, A. M., Watzky, M. A. & Finke, R. G. Protein aggregation kinetics, mechanism, and curve-fitting: A review of the literature. *Biochim. Biophys. Acta - Proteins Proteomics* **1794**, 375–397 (2009).

2. Roberts, C. J., Das, T. K. & Sahin, E. Predicting solution aggregation rates for therapeutic proteins: Approaches and challenges. *Int. J. Pharm.* **418**, 318–333 (2011).

3. Meisl, G. *et al.* Molecular mechanisms of protein aggregation from global fitting of kinetic models. *Nat. Protoc.* **11**, 252–72 (2016).

4. Morris, A. M., Watzky, M. A., Agar, J. N. & Finke, R. G. Fitting neurological protein aggregation kinetic data via a 2-step, minimal/"Ockham's razor" model: The Finke-Watzky mechanism of nucleation followed by autocatalytic surface growth. *Biochemistry* **47**, 2413–2427 (2008).

5. Singla, A., Bansal, R., Joshi, V. & Rathore, A. S. Aggregation Kinetics for IgG1-Based Monoclonal Antibody Therapeutics. *AAPS J.* **18**, 689–702 (2016).

6. Oliva, A., Llabrés, M. & Fariña, J. B. Fitting bevacizumab aggregation kinetic data with the Finke-Watzky two-step model: Effect of thermal and mechanical stress. *Eur. J. Pharm. Sci.* **77**, 170–179 (2015).

7. Boulet-Audet, M., Byrne, B. & Kazarian, S. G. High-throughput thermal stability analysis of a monoclonal antibody by attenuated total reflection FT-IR spectroscopic imaging. *Anal. Chem.* **86**, 9786–9793 (2014).

8. Morgan, G. J. & Kelly, J. W. The Kinetic Stability of a Full-Length Antibody Light Chain Dimer Determines whether Endoproteolysis Can Release Amyloidogenic Variable Domains. *J. Mol. Biol.* **428**, 4280–4297 (2016).

9. Borgia, M. B., Nickson, A. A., Clarke, J. & Hounslow, M. J. A mechanistic model for amorphous protein aggregation of immunoglobulin-like domains. *J. Am. Chem. Soc.* **135**, 6456–6464 (2013).

10. Cohen, S. I. A. *et al.* Proliferation of amyloid-β42 aggregates occurs through a secondary nucleation mechanism. *Proc. Natl. Acad. Sci. U. S. A.* **110**, 9758–9763 (2013).

11. Korson, L., Drost-Hansen, W. & Millero, F. J. Viscosity of water at various temperatures. *J. Phys. Chem.* **73**, 34–39 (1969).

12. Andrews, J. M., & Roberts, C. J. A Lumry-Eyring Nucleated Polymerization Model of Protein Aggregation Kinetics: 1. Aggregation with Pre-Equilibrated Unfolding. *J. Phys. Chem. B* **111,** 7897-7913 (2007).

13. Li, Y., Ogunnaike, B. A., & Roberts, C. J. Multi-Variate Approach to Global Protein Aggregation Behavior and Kinetics: Effects of pH, NaCl, and Temperature for a-Chymotrypsinogen A. *J. Pharm. Sci.* **99**, 645-662 (2010).

14. Baxter, R. J. Percus-Yevick Equation for hard spheres with surface adhesion. *J. Chem. Phys.* **49**, 2770 (1968).

15. Batchelor, G.K. Sedinentation in a dilute polyisperse system of interacting spheres. 1. General theory. *J. Fluid Mech.* **119**, 379-408 (1982).
